# The Arabidopsis anaphase‐promoting complex/cyclosome subunit 8 is required for male meiosis

**DOI:** 10.1111/nph.16014

**Published:** 2019-07-24

**Authors:** Rong‐Yan Xu, Jing Xu, Liudan Wang, Baixiao Niu, Gregory P. Copenhaver, Hong Ma, Binglian Zheng, Yingxiang Wang

**Affiliations:** ^1^ State Key Laboratory of Genetic Engineering and Ministry of Education Key Laboratory of Biodiversity Sciences and Ecological Engineering Institute of Plant Biology School of Life Sciences Fudan University Shanghai 200438 China; ^2^ Shanghai Chenshan Plant Science Research Center Chinese Academy of Sciences Chenshan Botanical Garden Shanghai 201602 China; ^3^ Key Laboratory of Plant Functional Genomics of the Ministry of Education Jiangsu Key Laboratory of Crop Genetics and Physiology/Co‐Innovation Center for Modern Production Technology of Grain Crops Yangzhou University Yangzhou 225009 China; ^4^ Department of Biology and the Integrative Program for Biological and Genome Sciences University of North Carolina at Chapel Hill Chapel Hill NC 27599‐3280 USA; ^5^ Lineberger Comprehensive Cancer Center University of North Carolina School of Medicine Chapel Hill NC 27599‐3280 USA; ^6^ Center for Evolutionary Biology Institutes of Biomedical Sciences School of Life Sciences Fudan University Shanghai 200433 China

**Keywords:** APC/C, *Arabidopsis thaliana*, chromosome segregation, meiosis, spindle assembly

## Abstract

- Faithful chromosome segregation is required for both mitotic and meiotic cell divisions and is regulated by multiple mechanisms including the anaphase‐promoting complex/cyclosome (APC/C), which is the largest known E3 ubiquitin‐ligase complex and has been implicated in regulating chromosome segregation in both mitosis and meiosis in animals. However, the role of the APC/C during plant meiosis remains largely unknown. Here, we show that Arabidopsis *APC8* is required for male meiosis.
- We used a combination of genetic analyses, cytology and immunolocalisation to define the function of AtAPC8 in male meiosis.
- Meiocytes from *apc8‐1* plants exhibit several meiotic defects including improper alignment of bivalents at metaphase I, unequal chromosome segregation during anaphase II, and subsequent formation of polyads. Immunolocalisation using an antitubulin antibody showed that APC8 is required for normal spindle morphology. We also observed mitotic defects in *apc8‐1,* including abnormal sister chromatid segregation and microtubule morphology.
- Our results demonstrate that Arabidopsis APC/C is required for meiotic chromosome segregation and that APC/C‐mediated regulation of meiotic chromosome segregation is a conserved mechanism among eukaryotes.

## Introduction

In meiosis, faithful chromosome segregation is crucial for ploidy stability during sexual life cycles (Zamariola *et al*., 2014b). Unlike mitosis, which has one round of sister chromatid segregation, meiosis has two rounds of segregation following a single round of premeiotic DNA replication. Meiosis I results in segregation of homologous chromosomes and meiosis II separates the sister chromatids. Several mechanisms ensure accurate segregation of chromosomes during meiosis, including the stepwise removal of cohesion from sister chromatid arms and centromeres (Rankin & Dawson, 2016; Bolanos‐Villegas *et al*., 2017). However, the mechanisms that regulate the attachment of spindle microtubules to kinetochores to ensure proper chromosome alignment and the onset of anaphase (Di Fiore *et al*., 2016; Ji *et al*., 2018) are not as well understood, particularly in plants including Arabidopsis.

The anaphase‐promoting complex/cyclosome (APC/C) is a multisubunit RING E3 ubiquitin ligase that regulates the progression of the eukaryotic cell cycle together with Cdc20 (its mitosis‐specific activator) or Cdh1 (its interphase‐specific activator) (Kashevsky *et al*., 2002; Pines, 2011; Sivakumar & Gorbsky, 2015; Severson *et al*., 2016). In plants, the APC/C consists of at least 11 core subunits, each of which is encoded by a single gene, except for APC3, which is encoded by two genes, *APC3a*/*CDC27a* and *APC3b*/*CDC27b*/*HOBBIT (HBT)*. The Arabidopsis APC/C activator *Cell Division Cycle 20.1* is required for bivalent alignment and chromosome segregation during meiosis (Niu *et al*., 2015), suggesting that the APC/C subunits may also have meiotic functions in plants. During meiotic maturation in mammalian oocytes, the APC/C is involved in virtually every stage (Homer, 2013) and disruption of either the APC/C or its substrates results in severe phenotypes. For example, deletion of the APC/C subunit APC2 gene causes major defects in fertility (McGuinness *et al*., 2009). In *C. elegans*, the APC/C gene *mat‐3/APC8* has been defined by temperature‐sensitive embryonic lethal alleles, which lead to defects in meiosis and mitosis (Garbe *et al*., 2004). In *Xenopus*, the APC is required for the second meiotic anaphase (Peter *et al*., 2001). In addition, genetic inactivation of APC/C components causes lethality in many species (Yu *et al*., 1998; Yamashita *et al*., 1999; Cullen *et al*., 2000; Bentley *et al*., 2002; Garbe *et al*., 2004; Pal *et al*., 2007; Jin *et al*., 2010). In plants, the APC/C core complex and its activators have been reported to play important roles in growth and development (Heyman & De Veylder, 2012; Wang *et al*., 2013; Guo *et al*., 2016). Although meiotic functions of the known plant APC/C subunits have not been reported, several meiotic regulators associated with APC/C have been identified. THREE DIVISION MUTANT1/MALE STERILE 5 (TDM1/MS5) regulates meiotic exit and is a putative meiotic APC/C component based on its shared structural similarities with the APC/C subunit CDC16/Cut9/APC6 (Cromer *et al*., 2012; Cifuentes *et al*., 2016). The direct interaction between TDM1with the APC/C activator CDC20.1 and the APC/C core component CDC27b (HOBBIT) further supports the hypothesis that TDM1 acts via the activation of the APC/C and/or by modifying its specificity. In addition, TDM1 also interacts with TAM/CYCA1;2 (an A‐type cyclin) and OSD1 (an APC/C inhibitor). Both proteins are required to prevent meiosis termination after meiosis I (Bulankova *et al*., 2010; d'Erfurth *et al*., 2010; Cromer *et al*., 2012). PATRONUS1 (PANS1) is required for the protection of centromere cohesion at meiosis II and interacts directly with CDC27b and CDC20.1 (Cromer *et al*., 2013; Zamariola *et al*., 2014a; Singh *et al*., 2015), suggesting that PANS1 could be an APC/C regulator or target.

An allele of Arabidopsis *APC8, apc8‐1*, was previously identified in a genetic screen for enhancers and/or suppressors of weak allele of *DCL1* (*dcl1‐14*), which is required for miRNA biogenesis. Analysis of *apc8‐1* showed that APC8 acts as a dual integrator mediating both microRNA‐dependent cyclin B1 transcription and its degradation during postmeiotic male gametophyte development (Zheng *et al*., 2011). More recently, the soybean *GmILPA1* gene encoding an APC8‐like protein, has been shown to have a role in the regulation of leaf petiole angle (Gao *et al*., 2017), suggesting multiple functions for APC8.

Here, we report that Arabidopsis APC8 is required for male meiosis. Analysis of chromosome dynamics in *apc8‐1* meiocytes shows irregular alignment of bivalents at metaphase I, improper segregation of sister chromatids at anaphase II, and the subsequent formation of polyads. Furthermore, *apc8‐1* exhibits abnormal microtubule organisation from metaphase I onward. These results provide evidence that the APC/C subunit plays an important role during meiotic chromosome segregation in plants.

## Materials and Methods

### Plant materials

All the *Arabidopsis thaliana* lines used in the study are in the ‘Columbia’ ecotype background. Wild‐type Col‐0 plants were used as controls. The *apc8‐1* allele is a point mutation (*c*. 925G>A) resulting in the substitution of a conserved amino acid, D309N, adjacent to the TPR domain. Transgenic complementation of *apc8‐1* with an APC8‐YFP fusion has been described previously (Zheng *et al*., 2011). PCR amplification using primers (Supporting Information Table S1) of a 251‐bp fragment including the SNP was used for genotyping. Digestion of the PCR products with *Apo*I followed by agarose gel analysis, yields bands of *c*. 190 bp and *c*. 60 bp using *apc8‐1* DNA and 251 bp using wild‐type DNA (Zheng *et al*., 2011). The other *apc* T‐DNA insertion alleles *apc1‐2*/at5g05560 (Salk_152651), *apc2‐2*/at2g04660 (Salk_010621), *apc4‐1/*at4g21530 (Salk_024729), *apc5‐2*/at1g06590 (Salk_036491), and *apc11*/at3g05870 (Salk_019654) were obtained from the Arabidopsis Biological Resource Center. All plants were grown in a glasshouse with a 16 h : 8 h, day : night photoperiod at 20°C with 70% humidity; 1–5 mm radicles harvested from germinated seeds were used for mitotic analyses.

### Real‐time PCR

Total RNA was extracted from inflorescences and 14‐d‐old seedlings using TRIzol reagent (Invitrogen). Each experiment included three biological replicates collected from independent plants and three technical replicates for each biological replicate. Reverse transcription was conducted using a Prime Script RT reagent kit with gDNA Eraser (cat. no. RR047A; TaKaRa), the products were then used as the template for quantitative PCR. Primers are listed in Table S1. PCR analysis was conducted using the Step One Plus Real‐Time PCR system (Applied Biosystems, Carlsbad, CA, USA) with iTaq Universal SYBR Green Supermix (cat. no. 72‐5124; Bio‐Rad). The cycle threshold (CT) value of the gene was normalised to CT of the internal control (*actin2*), and then the relative expression between samples was calculated using delta CT method (Schmittgen & Livak, 2008). The statistical significance of different gene expression levels was using the two‐tailed Student's *t*‐test.

### Cytological analyses

Inflorescences of Arabidopsis wild‐type and mutant plants were collected and fixed in a modified Carnoy's fixative (three ethanol : one acetic acid) for a minimum of 3 h. Pollen viability was analysed using a modified Alexander staining method by Peterson *et al*. (2010). Tetrad‐staged meiocytes were collected and stained with carbol fuchsin as previously described (Wang *et al*., 2014). Images of pollen and tetrads were obtained using a Zeiss Axio Imager microscope and processed using Adobe Photoshop CS6. Anthers at meiotic stages were collected from fixed inflorescences and used for cytological analyses. Chromosome spreading and fluorescence *in situ* hybridisation (FISH) analyses were performed as described previously (Wang *et al*., 2014).

### Immunolocalisation

Immunolocalisation of SYN1 and CENH3 were performed as described previously (Chelysheva *et al*., 2010), with modified antibody dilutions (below). For immunolocalisation of tubulin, inflorescences were fixed in 4 : 1 methanol and acetone mix. Selected materials were digested as previously described (Wang *et al*., 2014). Meiotic stage anthers were squashed and immobilised on glass slides coated with poly l‐Lys. After drying, the slides were incubated in phosphate‐buffered saline (PBS) with 1% Triton X‐100 for 1 h at room temperature, followed by blocking in PBS with 1.0 mM EDTA, 0.1% Tween‐20 for 1 h at room temperature. Samples were incubated at 4°C overnight with the primary antibodies. After rinsing in PBS buffer with 0.1% Tween‐20 three times, slides were incubated with secondary antibodies at 37°C for 2 h and washed three times in PBS. Immunolocalisation samples were then stained with antifade medium containing 2 mg ml^−1^ DAPI (Vector Laboratories, Burlingame, CA, USA). Observations were recorded using a Zeiss Axio Imager fluorescence microscope and processed using Adobe Photoshop CS6. The dilution of primary antibodies was 1 : 100 for CENH3 (ab72001; Abcam, Cambridge, MA, USA), 1 : 200 for SYN1 (Wang *et al*., 2012a), SMC3 (Wang *et al*., 2016) and ASY1 (Wang *et al*., 2012a), and 1 : 500 for α‐tubulin (Beyotime Biotech, Shanghai, China). All secondary antibodies labelled with fluorophore were diluted 1 : 1000.

### Yeast two‐hybrid assay

The full‐length cDNA of *AtAPC8, AtAPC10* and *AtCDC20.1* were obtained from reverse transcription PCR (RT‐PCR) of mRNA extracted from inflorescences of Arabidopsis plants. The PCR product of *AtAPC8* was cloned into the pEASY‐Blunt Cloning Vector (Transgene Biotech Co., Beijing, China; CB101‐01). The product of the APC8‐1 point mutation was amplified from apc8‐1 cDNA to produce a clone of the mutant allele designated APC8‐D309N. Primers are listed in Table S1 and Vazyme Super‐Fidelity DNA polymerase was used (Vazyme Biotech Co., Nanjing, China; P515‐01). The primers used to amplify AtAPC10 (*Nde*I/*Eco*RI) and AtCDC20.1 (*Nde*I/*Bam*HI) included engineered restriction enzyme sites. The amplicons were digested with the corresponding enzymes and ligated into the pGADT7 vector using T4 DNA ligase (Thermo Scientific Biotech Co., Waltham, MA, USA; EL0014). PCR products of APC8 and APC8‐D309N were purified and ligated into the pGBKT7 vector using NOVOREC PLUS DNA recombinase (Novoprotein Biotech Co., Suzhou, China; NR001A). The recombinant plasmids were verified by DNA sequencing and transformed into Y187 yeast strain (pGADT7) and Y2H Gold yeast strain (pGBKT7), accordingly. Transformants were mated on YPDA (yeast, peptone, dextrose, adenine) medium for 48 h, and selected on double dropout medium without leucine and tryptophan (DDO) plates for 48 h as well as the quadruple dropout medium lacking leucine, tryptophan, histidine, and adenine, and with X‐α‐Gal (g) (QDO) for 6 d.

### Structural modelling

The predicted structural model of APC8 was developed using the Phyre2 server (http://www.sbg.bio.ic.ac.uk/phyre2/html/page.cgi?xml:id=index) (Kelley *et al*., 2015) based on the structure of human CDC23 (PDB code: 4UI9) (Schreiber *et al*., 2011).

## Results

### Arabidopsis APC8 is required for meiosis

**Figure 1:**
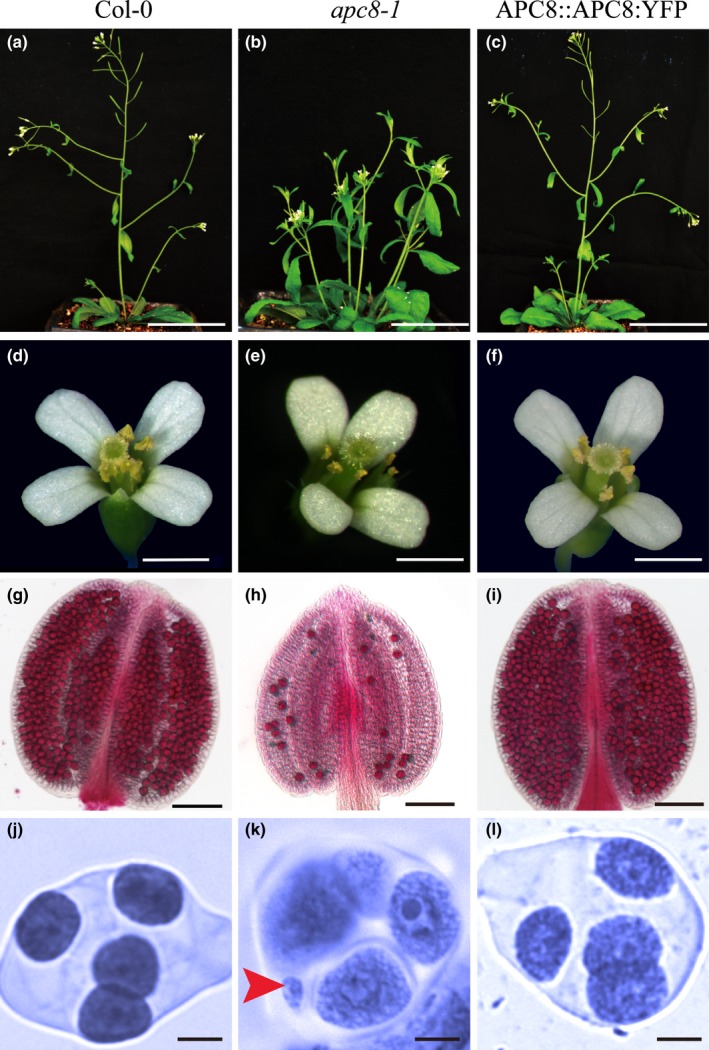
Phenotypes of wild‐type (WT), *apc8‐1* and APC8‐complemented Arabidopsis. WT (a), *apc8‐1* (b), and APC8‐complemented plants (c). Flowers of WT (d), *apc8‐1* (e), and APC8‐complemented plants (f). Alexander staining of anthers to assay pollen viability in WT (g), *apc8‐1* (h), and APC8‐complementated plants (i). In total, six independent plants were analysed. Viable and inviable pollen grains are stained in red and dark green, respectively. Tetrads stained with carbol fuchsin were prepared from WT (j), *apc8‐1* (k), and APC8‐complementated plants (l), respectively. Arrowhead indicates micronucleus. Bars: (a–c) 5 cm; (d–f) 1 mm; (g–i) 100 µm; (j–l) 25 µm.

The Arabidopsis *apc8‐1* point mutant is hypomorphic and has pleiotropic phenotypes, including abnormal development of the meristem, leaves and shoots, as well as reduced fertility with short siliques and mature pollen with no or single sperm‐like cells (Zheng *et al*., 2011), indicating that APC8 plays an important role in vegetative and reproductive development. Although the *apc8‐1* mutant is defective in vegetative growth (Fig. 1a–c), its floral organs are generally similar to wild‐type organs (Fig. 1d–f). Consistent with the observed reduced fertility, *apc8‐1* anthers have reduced pollen production compared with wild‐type (Fig. 1d–f). Furthermore, Alexander staining of anthers showed that *apc8‐1* has fewer viable pollen grains (25 ± 6/anther, *n* = 30) (Fig. 1h) than in either wild‐type (525 ± 41/anther, *n* = 15) or the APC8‐YFP complementation line (498 ± 38/anther, *n* = 15) (Fig. 1g,i). At the tetrad meiocyte stage (*n* = 30 per genotype), the four microspores in wild‐type and APC8‐YFP were uniform in size (Fig. 1j,l), whereas the polyads in *apc8‐1* had > 4 microspores of unequal size (Fig. 1k), further supporting a meiotic defect in *apc8‐1*. Finally, *apc8‐1* had an average of 12.8 seeds per silique (*n* = 11) compared with wild‐type with an average of 51.6 (*n* = 13) (Fig. S1b; Student's *t*‐test, *P* < 0.01). To verify that the embryo lethal phenotype of the null *apc8* alleles is not unusual, we obtained T‐DNA insertion alleles for five of the 11 other APC/C subunits (*APC1*,* APC2*,* APC4*,* APC5* and *APC11*). PCR analysis of the respective alleles in segregating populations from each line failed to identify any homozygous mutant plants, supporting the essential role of most APC/C subunits. Consistent with this observation, the heterozygotes *apc1‐2* ^+/−^ (33.3, *n* = 5), *apc2‐2* ^+/−^ (30.4, *n* = 5), *apc4‐1* ^+/−^ (35.2, *n* = 5), and *apc11* ^+/−^ (25.6, *n* = 5), have fewer viable seeds in their siliques compared with wild‐type (51.6, *n* = 13) (Fig. S1). The segregation ratio of normal vs dead seeds for *apc1‐2* ^*+/−*^, *apc2‐2* ^*+/−*^ and *apc4‐1* ^*+/−*^ is consistent with a 3 : 1 ratio, but lower for *apc11* ^*+/−*^. Together, these data support the essential role of APC/C subunits in regulating the cell cycle and that APC8 is also required for meiosis.

The *apc8‐1* mutant is a point mutation (925G>A). To examine the expression of *APC8* in this mutant, we performed qRT‐PCR analyses using seedlings and inflorescences from wild‐type and *apc8‐1*. The expression of APC8 showed no significant difference in *apc8‐1* compared with wild‐type in leaf tissue (Fig. S2a), but was significantly higher in *apc8‐1* inflorescences (*P* < 0.001) relative to wild‐type (Fig. S2a), suggesting that the point mutation in *APC8* had a positive effect on its expression in inflorescences. Previous studies have demonstrated that AtTDM1 and AtOSD1 are an APC/C component and inhibitor, respectively (Cromer *et al*., 2012; Cifuentes *et al*., 2016), and AtPANS1 is an APC regulator (Cromer *et al*., 2013; Zamariola *et al*., 2014a; Singh *et al*., 2015). We analysed the expression of these three genes in inflorescences of wild‐type, *apc8‐1,* APC8‐complementated plants, and *AtTDM1* and *AtOSD1* expression showed a significant elevation in *apc8‐1* (*P* < 0.01) and APC8‐YFP plants (*P* < 0.05) compared with wild‐type (Fig. S2b), while the AtPANS1 expression was unaltered among the samples examined (Fig. S2b). Taken together, these results suggested that APC/C and its components are aberrantly activated in the *apc8‐1* mutant and this increased activity triggers the need for more AtOSD1 to facilitate proper inhibition.

### AtAPC8 ensures proper bivalent alignment and subsequent accurate segregation of sister chromatids

**Figure 2:**
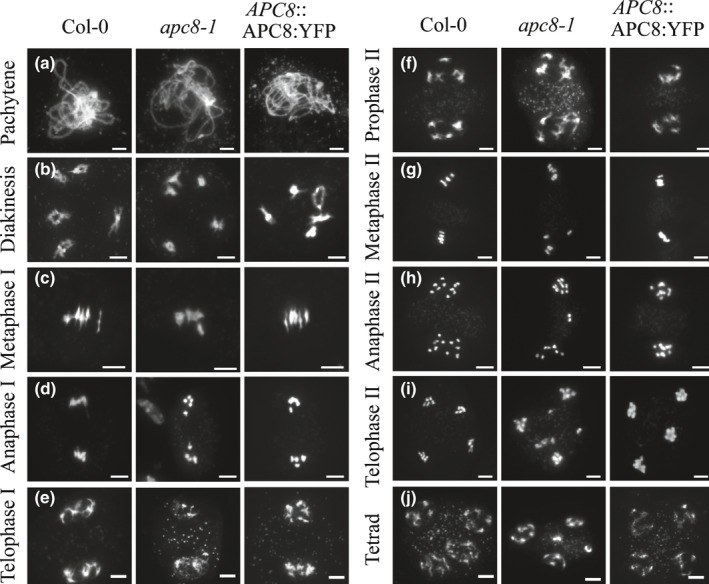
Meiotic chromosome configurations of wild‐type (WT), *apc8‐1* and APC8‐complemented Arabidopsis. In each panel, left column, WT; middle column, *apc8‐1*; right column, APC8‐complemented chromosome spreads. (a) pachytene; (b) diakinesis; (c) metaphase I; (d) anaphase I; (e) telophase I; (f) prophase II; (g) metaphase II; (h) anaphase II; (i) telophase II; and (j) tetrad stage. All pictures were taken with a ×100 objective using a fluorescence microscope (Zeiss Axio Imager). Bars, 5 μm.

To investigate the meiotic defects in *apc8‐1* in detail, chromosome spreads from male meiocytes stained with 4′,6‐diamidino‐2‐phenylindole (DAPI) were assessed. Wild‐type and *apc8‐1* chromosomes have similar thread‐like structures through pachytene (Fig. 2a) that condense into five bivalents at diakinesis (Fig. 2b). At metaphase I the five bivalents, pushed by the spindle, are aligned along the equator in wild‐type (Fig. 2c), whereas *c*. 36.7% (*n* = 30) of *apc8‐1* meiocytes have disorderly in bivalent alignment (Fig. 2c), similar to the phenotype of the previously identified *cdc20.1* mutant (Niu *et al*., 2015). Even though some bivalents are not aligned at the metaphase I plate, the homologues still successfully segregate at anaphase I (Fig. 2d). However, some lagging chromosomes are observed, indicating aberrant segregation dynamics. Nonetheless, at later stages, no obvious chromosome behaviour defects were observed from telophase I to prophase II (Fig. 2e,f). Similar to metaphase I, we also observed a misalignment of chromosomes at metaphase II plate in one or two poles in the *apc8‐1* mutant in 40% (*n* = 30) of meiocytes (Fig. 2g). However, *c*. 85% (*n* = 30) of anaphase II meiocytes have uneven separation of sister chromosomes (Fig. 2h). As a consequence, *c*. 90% of *apc8‐1* meiocytes (*n* = 35) form abnormal tetrads or polyads (Fig. 2j). As a control, the APC8‐complementated plants exhibited similar meiotic chromosome behaviours to that of wild‐type at all the observed stages (Fig. S3). Because *apc8‐1* is a weak mutant allele of APC8, these analyses are likely to underestimate the role of APC8 in meiosis. Therefore, these data provide evidence that APC8 is required for proper meiotic chromosome alignment and segregation.

The abnormal vegetative development in *apc8‐1* indicates a possible defect in mitotic cell division. To test this possibility, we examined mitotic chromosome spreads from root tip cells harvested from wild‐type, *apc8‐1* and APC8‐complementated plants. We observed several types of mitotic anomalies in *apc8‐1* cells including 3.03% (*n* = 99) with abnormally aligned sister chromatids at metaphase (Fig. S4f), 11.9% (*n* = 42) with lagging chromosomes at anaphase (Fig. S4fg), and 12.5% (*n* = 24) at telophase (Fig. 4h). By contrast, no abnormal chromosome configurations were observed in mitotic cells from wild‐type or APC8‐complementated plants (Figs S4a–d, 4i–l). These data support the idea that the abnormal vegetative development observed in *apc8‐1* is at least partially due to mitotic chromosome segregation defects.

### Cohesin complex and chromosome axis protein ASY1 appears to be unaffected in *atapc8‐1*

**Figure 3:**
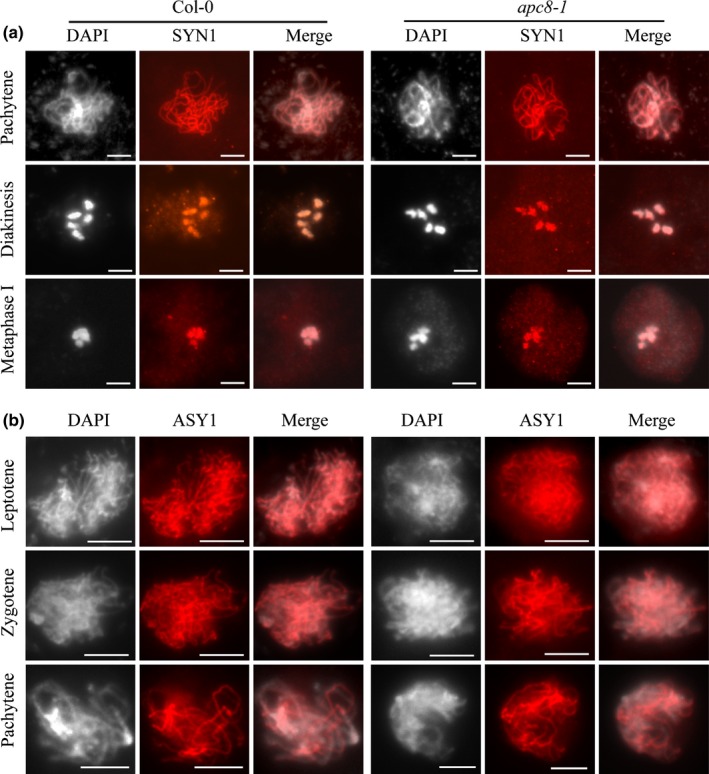
Immunolocalization of AtSYN1 and AtASY1 in wild‐type (WT) and *apc8‐1* Arabidopsis. (a) SYN1 and (b) ASY1 were immunolocalised to chromosomes from zygotene to metaphase I in WT and *apc8‐1*. Grey images show DAPI‐stained chromosomes; red colour along the chromosomes indicates SYN1 and ASY1 signals. Thirty cells for each stage were observed. All pictures were taken with a ×100 objective using a fluorescence microscope (Zeiss Axio Imager). Bars, 5 μm.

To ensure successful chromosome segregation, both mitosis and meiosis require proper assembly of cohesin complexes between the sister chromatids (Klein *et al*., 1999; Watanabe, 2004; Brooker & Berkowitz, 2014). The timely removal of cohesin complexes by separase depends on the degradation of cyclin B1 by the APC/C (Hellmuth *et al*., 2015; Sivakumar & Gorbsky, 2015). The misalignment of meiotic bivalents in *apc8‐1* resembles the phenotype observed in *cdc20.1* meiocytes (Niu *et al*., 2015) in which cohesin establishment is normal. We analysed the distribution of Arabidopsis SYN1 (Cai *et al*., 2003), a homologue of the yeast meiosis‐specific cohesin REC8 (Meiotic recombination‐deficient 8), in wild‐type and *apc8‐1* meiotic chromosome spreads. SYN1in wild‐type pachytene meiocytes is distributed in a linear pattern along chromosomes (Fig. 3a, *n* = 17), consistent with its typical distribution characteristics (Chelysheva *et al*., 2010). SYN1 continues to colocalise with chromosomes through diakinesis (wild‐type, *n* = 18; *apc8‐1*,* n* = 23) and metaphase I (wild‐type, *n* = 15; *apc8‐1*,* n* = 30). No obvious differences are seen in *apc8‐1* (Fig. 3a, *n* = 30) at any stage. This finding suggests that loading of SYN1 onto chromosomes is unaffected in *apc8‐1*, consistent with the *cdc20.1* phenotype (Niu *et al*., 2015). To validate the lack of a cohesin defect in *apc8‐1*, we examined another cohesin component, SMC3 (Lam *et al*., 2005), and found that the SMC3 signal exhibited no obvious difference at zygotene, pachytene, or diakinesis (Fig. S5) between wild‐type and *apc8‐1* (*n* = 21), confirming that cohesin establishment is normal in *apc8‐1*. However, due to the limited sensitivity of the technique and/or very low protein levels, it was not possible to examine SYN1 or SMC3 during meiosis II. Therefore, whether the removal of cohesin at anaphase I and/or II is regulated by APC8 remains an open question. We also examined the localisation of the chromosome axis component ASY1 (Armstrong *et al*., 2002) in *apc8‐1* and found no difference compared with wild‐type (Fig. 3b; *n* = 30), suggesting that the early assembly of the chromosome axis appears to be normal.

### AtAPC8 is required for the normal alignment of the centromere and the kinetochore protein AtCENH3 in meiosis

**Figure 4:**
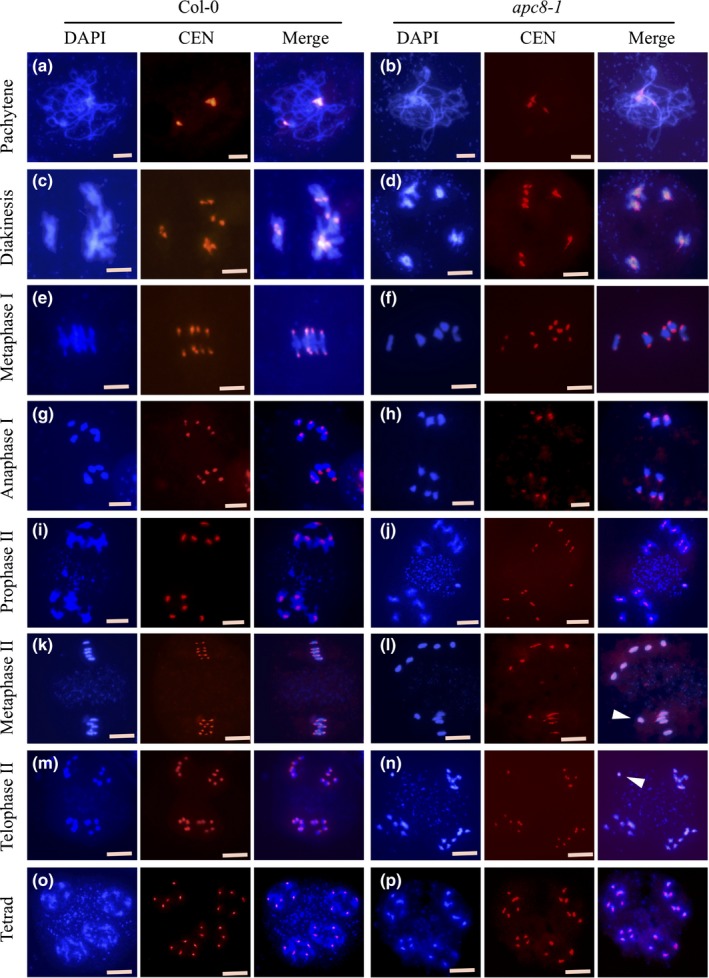
FISH analysis of meiotic chromosomes in wild‐type (WT) and *apc8‐1* Arabidopsis using a centromere probe. Chromosomes spreads were hybridised with a 180‐bp repeat centromere DNA probe. Blue images show chromosomes stained with DAPI; red spots show DNA signals at centromeres. (a, b) pachytene, (c, d) diakinesis, (e, f) metaphase I, (g, h) anaphase I, (i, j) prophase II, (k, l) metaphase II, (m, n) telophase II, and tetrad stages (o, p), respectively. Arrowheads in row (d) show centromeres in a pair of lagging chromosomes in the *apc8‐1* mutant. Arrows in (l, n) shows misaligned chromosomes at metaphase II in *apc8‐1*. All pictures were taken with a ×100 objective using a fluorescence microscope (Zeiss Axio Imager). Bars, 5 μm.

Given the normal establishment of cohesion in *apc8‐1*, the misalignment of bivalents at metaphase I may indicate a defect in orientating the chromosomes on the spindle. To test this hypothesis, we analysed chromosome spreads using FISH with a probe corresponding to the 180‐bp centromere repeat. Chromosome configurations were indistinguishable between wild‐type and *apc8‐1* at diakinesis (Fig. 4a–d). At metaphase I, *c*. 95% (*n* = 30) of wild‐type meiocytes had five pairs of centromere signals, with each pair distributed to both sides of one bivalent (Fig. 4e). By contrast, *c*. 40% (*n* = 30) of *apc8‐1* meiocytes had asymmetrically aligned centromere signals (Fig. 4f), this is consistent with the misalignment observed with DAPI‐stained chromosome spreads (Fig. 2c). At anaphase I, centromere signals in wild‐type and *apc8‐1* (*n* = 30) formed two clusters, each with five centromeres (Fig. 4g–h), with no obvious differences. During meiosis I, in wild‐type the two sister chromatids are mono‐oriented and the sister kinetochores are fused and co‐migrate during segregation of homologous chromosomes (Sarangapani *et al*., 2014). By comparison, the misaligned bivalents in *apc8‐1* meiocytes suggest that the kinetochore orientation on the spindle may be defective.

**Figure 5:**
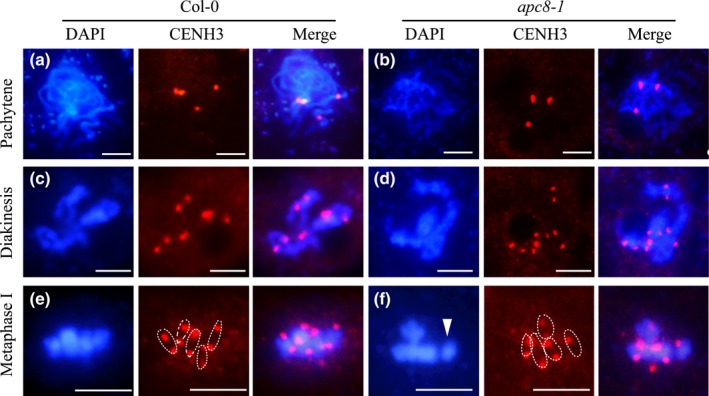
Immunolocalization of AtCENH3/CENP‐A in wild‐type (WT) and *apc8‐1* Arabidopsis. CENH3 was immunolocalised to meiotic chromosomes during pachytene (a, b), diakinesis (c, d), and metaphase I (e, f) using an anti‐HTR12 (CENH3) antibody. Blue images show DAPI‐stained chromosomes; red spots are CENH3 signals. White dotted lines outline the CENH3 signal in a bivalent (the middle panels of e and f). Arrowhead in (f) marks the bivalent with an abnormal CENH3 signal. All pictures were taken with a ×100 objective using a fluorescence microscope (Zeiss Axio Imager). Bars, 5 μm.

Interestingly, at prophase II, wild‐type meiocytes have five concentrated centromeres at both poles (Fig. 4i), but in *apc8‐1* meiocytes (*n* = 30) the pairs of centromeric signals are slightly separated (Fig. 4j), suggesting a defect in the protection of centromeric cohesin complexes between sister chromatids in meiosis II. At metaphase II, wild‐type chromosomes are aligned (Fig. 4k), while *apc8‐1* (*n* = 30) chromosomes are misalignment (Fig. 4l), providing additional evidence that APC8 is required for chromosome segregation in meiosis II. Finally, abnormal chromosome segregation in *apc8‐1* (Fig. 4n), compared with wild‐type (Fig. 4m), leads to the production of dysfunctional tetrads with four nuclei containing various amounts of DNA (Fig. 4p), or to polyads. These results are consistent with APC8 playing a role in meiotic chromosome segregation and proper interaction of the spindle microtubules and sister kinetochores at metaphase I. To explore this further we examined the immunolocalisation of the kinetochore marker protein CENH3 (CENP‐A) and found that CENH3 signals are indistinguishable between wild‐type and *apc8‐1* at pachytene and diakinesis (Fig. 5a–d). At metaphase I, we detected CENH3 signals at two sides of the well aligned bivalents in wild‐type (Fig. 5e). By contrast, in *apc8‐1* the distribution of CENH3 appears asymmetric (Fig. 5f), and *c*. 40% of cells (*n* = 30) in have one separated bivalent, which corresponds to the misalignment of bivalents at metaphase I. Based on these observations, we hypothesise that APC8 plays an important role in the normal sister kinetochore mono‐orientation during meiosis.

### Formation of normal spindle morphology during meiosis and mitosis requires AtAPC8

**Figure 6:**
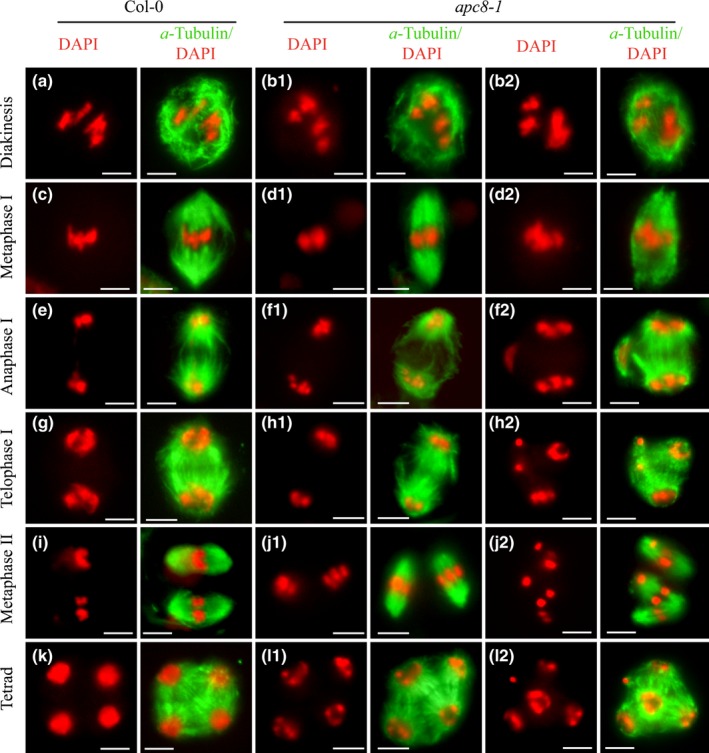
Immunolocalization of microtubules in meiocytes in wild‐type (WT) and *apc8‐1* Arabidopsis. Microtubule organisation from diakinesis to tetrad in WT (a, c, e, g, i and k), normal microtubule arrangement (b1, d1, f1, h1, j1 and l1), and abnormal microtubule arrangement (b2, d2, f2, h2, j2 and l2) in *apc8‐1*. Microtubules were immunolocalised with an antitubulin antibody. Chromosomes were stained with DAPI. All pictures were taken with a ×100 objective using a fluorescence microscope (Zeiss Axio Imager). Bars, 5 μm.

The spindle is required for chromosome movement and segregation during mitosis and meiosis (Nagasaka *et al*., 2011; Duro & Marston, 2015), and accurate chromosome segregation is accomplished by proper attachment of chromosomes to the spindle microtubules via the kinetochore (Chan *et al*., 2005; Severson *et al*., 2016). We hypothesise that the aberrant chromosome segregation in *apc8‐1* is caused by defects in establishing a normal spindle. To test this we examined microtubule assembly. At diakinesis in both wild‐type and *apc8‐1* we found no obvious differences in microtubule arrangement. In both cases the microtubules were organised in a perinuclear arrangement (Fig. 6a,b). At metaphase I, the microtubules in wild‐type were rearranged into a bipolar spindle with sharply focused poles (Fig. 6c), whereas the *apc8‐1* spindles (*n* = 30 cells) appeared twisted or narrow (87%), or had misaligned chromosomes (7%) (Fig. 6d1,d2). During anaphase I, the wild‐type spindle lengthened along the polar axis, pulling the chromosomes to separate them (Fig. 6e) and, at metaphase II, the microtubules formed two smaller spindles (Fig. 6i). By contrast, in *apc8‐1* the main spindle had two large unfocused poles and, along the side of the main spindle, 10% (*n* = 30) had an unusual small spindle‐like structure around a chromosome (Fig. 6f2). Subsequently, 10% (*n* = 30) had a multipolar structure that formed at telophase I (Fig. 6h2) and at metaphase II, and 30% of cells (*n* = 30) formed two spindles in addition to a mini‐spindle that harbored the misaligned chromosomes (Fig. 6j2). Finally, wild‐type formed tetrads with microtubules surrounding the four nuclei (Fig. 6k), whereas in *apc8‐1* 40% (*n* = 30) of meioses resulted in polyads with multipolar spindles (Fig. 6l2).

We also observed the morphology mitotic spindles and found that during prophase microtubules were in a perinuclear arrangement in both wild‐type and *apc8‐1* (Fig. S6a,b). At metaphase, regular spindles were observed in wild‐type (Fig. S6c); however, in *apc8‐1* abnormal spindles with misaligned chromosomes were observed and a minispindle‐like structure was found next to the abnormal main spindle (Fig. S6d, arrowhead). Subsequently, free chromosomes were observed in both anaphase and telophase in *apc8‐1* (Fig. S6f,h). Taken together, these results indicated that APC8 is required for normal microtubule organisation and spindle assembly. Furthermore, the defective microtubule organisation and dysfunctional spindles observed during mitosis and meiosis are likely to be the primary cause of the metaphase I chromosome misalignment and aberrant segregation at meiosis II in *apc8‐1*.

### The *atapc8‐1* point mutation disrupts interaction with AtCDC20.1

CDC20.1, an APC/C activator, interacts with APC3s, APC8 and APC10 (Fülöp *et al*., 2005; Perez‐Perez *et al*., 2008; Kevei *et al*., 2011; Qiao *et al*., 2016). To investigate whether *apc8‐1* has altered protein interactions, we used yeast two‐hybrid assays and found that APC8 interacts with APC10, CDC20.1 and itself, while APC8^D309N^ still interacts with APC10 and itself, but not with CDC20.1. (Fig. S7a). APC8 self‐interaction is consistent with homodimer formation of the human homologue (Schreiber *et al*., 2011). Previous analyses demonstrated that the TPR domain of the APC/C of various subunits is required for protein−protein interaction (Kevei *et al*., 2011; Qiao *et al*., 2016). We modelled the structure of the full‐length APC8 using the SWISS‐MODEL (https://www.swissmodel.expasy.org) protein structure prediction tool (Waterhouse *et al*., 2018). Protein sequence alignment showed that Arabidopsis APC8 (61−562 aa) has *c*. 60% similarity to human CDC23 (47−547 aa) (Fig. S7b), which has four electron microscopy structures deposited in the Protein Structure Database (PDB code: 4UI9, 5A31, 5KHU and 5G04). We built the predicted APC8 model using the 4UI9 structure as template (Fig. S7c,d), and found that D309 in APC8 corresponds to D301 in CDC23 while, in the 4UI9 structure, D301 and Y364 are closed to each other. We therefore speculated that the amino acid substitution in APC8^D309N^ may affect the interaction between D309 and Y372 (corresponding to Y364 in human CDC23), and interfere with the TPR domain's ability to facilitate protein−protein interaction.

## Discussion

### AtAPC/C is indispensable for normal development and meiosis in Arabidopsis

As an essential E3 ubiquitin protein ligase that mediates the mitotic transition, the APC/C is highly conserved among eukaryotes (Peters, 2002). Mutations in APC/C subunits are severely detrimental in vertebrates and plants, including Arabidopsis (Capron *et al*., 2003; Kwee & Sundaresan, 2003; McGuinness *et al*., 2009; Eloy *et al*., 2011; Wang *et al*., 2012b; Wang *et al*., 2013; Zhang *et al*., 2014). In Arabidopsis, functional characterisation of the APC/C subunits APC1, APC2, APC4, APC6, APC8, and APC13 has demonstrated their indispensability for female gametogenesis and embryogenesis (Capron *et al*., 2003; Kwee & Sundaresan, 2003; Eloy *et al*., 2011; Zheng *et al*., 2011; Wang *et al*., 2012b, 2013). Genetic screening for mutants with defects in zygote development identified APC11 and the null allele of this gene as embryonic lethal (Guo *et al*., 2016). The probable function of the APC/C during mitotic cell division is to regulate cyclin B degradation. However, whether the APC/C subunit is required for meiotic cell division has not been previously reported. Here, we used several approaches to analyse the meiotic phenotypes of a previously identified weak allele of *apc8‐1* (Zheng *et al*., 2011) and found that, in addition to the previously reported phenotypes, the reduced fertility in *apc8‐1* is, at least partially, caused by meiotic defects. We provide several lines of cytological evidence to support this conclusion. First, unlike wild‐type tetrads, the *apc8‐1* meiocytes produced polyads, indicative of unequal meiotic chromosome segregation. Second, chromosome behaviour assessed by DAPI staining showed that misaligned chromosomes were observed at both mitosis and meiosis metaphase I in *apc8‐1*, further supporting a defect in chromosome segregation. Third, we also found that chromosome segregation defects in *apc8‐1* were likely to be due to abnormal spindle orientation rather than the loading or removal of cohesin. Taken together, our results demonstrated that APC8 is essential for meiosis in plants.

### A potential role for AtAPC8 in meiotic chromosome segregation

**Figure 7:**
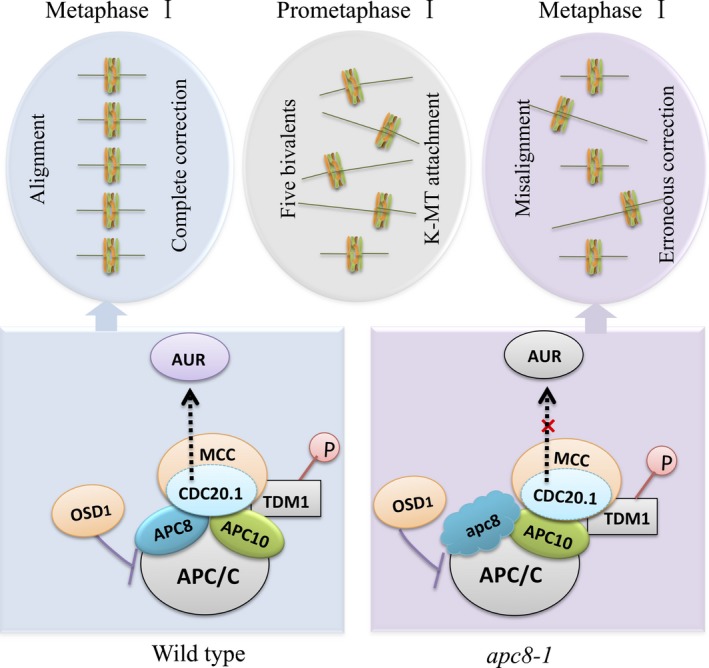
A model showing the potential role of AtAPC8 in bivalent alignment during prometaphase I. In wild‐type, at meiotic prometaphase I, the initial attachment of kinetochores to microtubules (K‐MT) is likely asynchronous and stochastic. Erroneous attachment activates a robust SAC signal, in which a SAC effector−MCC complex inhibits APC/C activity via regulation of CDC20.1. The correction of erroneous K‐MT attachments is mediated by the Aurora kinase (AUR) promoted by CDC20.1. Once all corrections are implemented, the spindle aligns chromosomes at the equatorial plate. In *apc8‐1*, APC8^D309N^ does not interact with CDC20.1, which comprises AUR ability to correct K‐MT attachments. As a consequence, the bivalents are not well aligned at metaphase I.

Based on our results and previous findings, we present a model to illustrate the role of the APC/C and associated factors in bivalent alignment during meiotic prophase I (Fig. 7). During wild‐type prometaphase I of meiosis, kinetochores can attach to microtubules (K‐MT) normally or erroneously, and erroneous attachments can be corrected by a complex system involving the mitotic checkpoint complex (MCC), Aurora kinase (AUR1) and/or the APC/C complex, as well as its associated factors OSD1 and TDM1. During attachment correction, the ability of CDC20.1 to activate APC/C is partially inhibited by MCC and/or OSD1 or both. Under these conditions CDC20.1 promotes AUR1 correction of erroneous K‐MT attachment. In wild‐type, once all correction is implemented, the spindle aligns chromosomes at the equatorial plate at metaphase I. In *apc8‐1*, APC8^D309N^ does not interact with CDC20.1 and erroneous spindle attachments persist or their correction is delayed, leading to chromosome misalignment at metaphase I (Fig. 7). Previous studies demonstrated that OSD1 is an APC/C inhibitor, while TDM1 is present throughout meiosis and its activity must be inhibited during meiosis I (Cromer *et al*., 2012; Cifuentes *et al*., 2016). Our qRT‐PCR data show that the expression of both genes is elevated in *apc8‐1* compared with wild‐type, indicating that there may be a feedback mechanism between APC/C and its regulators.

Although we observed *c*. 40% abnormal metaphase I alignment of bivalents in *apc8‐1*, this does not lead to uneven segregation of homologues at anaphase I. A possible explanation for this observation is that spindle assembly checkpoint (SAC) activity may be compromised in *apc8‐1*, thereby delaying meiotic progression. Furthermore, the meiosis I *apc8‐1* defects cannot explain the *c*. 95% frequency of abnormal tetrads at telophase II. It is plausible that APC8 may have a role in chromosome segregation during meiosis II, but this requires further investigation.

### Conservation and divergence of APC/C activity in meiosis among species

The APC/C is highly conserved with 11 subunits in both plants and animals. Analysis of APC/C subunit null alleles that show they are essential in all species tested ranging from fungi to mice (Peters, 2006), suggested a conserved function in the cell cycle. Because APC/C subunits are essential, much of our understanding of their role in chromosome segregation during meiosis has been derived from studies focusing on APC/C co‐activators or regulators (Cooper *et al*., 2000; Pesin & Orr‐Weaver, 2008; Kimata *et al*., 2011; Okaz *et al*., 2012; Holt *et al*., 2014). These regulators include yeast Ama1, a member of the Cdc20 protein family that directs meiosis I, but not meiosis II, APC/C function (Cooper *et al*., 2000; Okaz *et al*., 2012). In fission yeast, Mes1 interacts with two co‐activators, Slp1/Cdc20 and Fzr1/Mfr1, in a different manner to control the activity of the APC/C required for the meiosis I/meiosis II transition (Kimata *et al*., 2011). In mammals, the APC/C^FZR1^ meiosis‐specific activity regulates spermatogonial proliferation as well as early prophase I in both male and female germ cells (Holt *et al*., 2014). Although FZR is not a homologue of Ama1, it activates the APC/C in a similar manner when CDK1 activity is low (Peters, 2006). Additionally, APC/C^FZR1^ regulates a wide range of cell‐cycle events (Holt *et al*., 2014), suggesting a conservation of function utilising different proteins in different species. A similar conservation of function using different protein players has been recently described for the Arabidopsis SAC compared with animals and yeast (Komaki & Schnittger, 2017). The essential function of APC/C subunits in plants has limited our understanding of their function during chromosome segregation. Evidence suggests that Arabidopsis TDM1 is an APC/C component and is required for the termination of meiosis II (Cromer *et al*., 2012; Cifuentes *et al*., 2016). OSD1 promotes entry into the second meiotic division, likely to be through inhibition of APC/C activity (Cromer *et al*., 2012). Here we reported the role of APC8 in bivalent alignment based on the analysis of a weak mutant. Given the conservation of APC/C function across wide species boundaries, APC8 may play a similar role in other species, but this needs to be validated in future studies.

## Author contributions

R‐YX performed immunolocalisation of microtubules, analyses of CENH3 and other assessments; JX performed real‐time PCR, yeast two‐hybrid assays; R‐YX, B‐XN, JX and L‐DW performed FISH, SYN1 analysis and chromosome spreading; B‐LZ provided the mutant line *apc8‐1* and revised the manuscript; Y‐XW guided the study and helped in designing the experiments; R‐YX, JX, HM, GPC and Y‐XW contributed to data interpretation and writing the manuscript. R‐YX, JX and LW contributed equally to this work.

## Supporting information

Please note: Wiley Blackwell are not responsible for the content or functionality of any Supporting Information supplied by the authors. Any queries (other than missing material) should be directed to the *New Phytologist* Central Office.

**Fig. S1** Silique images and seed numbers in wild‐type, *atapc* mutants and the APC8‐complementated plants.
**Fig. S2** Relative transcript levels of *AtAPC8*,* AtOSD1*,* AtTDM1* and *AtPANS1* in wild‐type (Col‐0), *atapc8‐1* and APC8‐complementated plant.
**Fig. S3** FISH analysis of meiotic chromosomes in APC8‐complementated plants using a centromere probe.
**Fig. S4** Mitotic chromosome behaviours in wild‐type, *atapc8‐1* and APC8‐complementated plants.
**Fig. S5** Immunolocalisation of AtSMC3 in wild‐type and *atapc8‐1*.
**Fig. S6** Immunolocalisation of microtubules during mitosis in wild‐type and *atapc8‐1* mutant.
**Fig. S7** Examination of protein−protein interaction by yeast two‐hybrid assay and modelling the APC8 protein structure.
**Table S1** Primers used in this study.

## References

[nph16014-bib-0001] Armstrong SJ , Caryl AP , Jones GH , Franklin FC . 2002 Asy1, a protein required for meiotic chromosome synapsis, localizes to axis‐associated chromatin in Arabidopsis and Brassica. Journal of Cell Science 115: 3645–3655. 12186950 10.1242/jcs.00048

[nph16014-bib-0002] Bentley AM , Williams BC , Goldberg ML , Andres AJ . 2002 Phenotypic characterization of Drosophila ida mutants: defining the role of APC5 in cell cycle progression. Journal of Cell Science 115: 949–961. 11870214 10.1242/jcs.115.5.949

[nph16014-bib-0003] Bolanos‐Villegas P , De K , Pradillo M , Liu D , Makaroff CA . 2017 In favor of establishment: regulation of chromatid cohesion in plants. Frontiers in Plant Science 8: 846. 28588601 10.3389/fpls.2017.00846 PMC5440745

[nph16014-bib-0004] Brooker AS , Berkowitz KM . 2014 The roles of cohesins in mitosis, meiosis, and human health and disease. Methods in Molecular Biology 1170: 229–266. 24906316 10.1007/978-1-4939-0888-2_11 PMC4495907

[nph16014-bib-0005] Bulankova P , Riehs‐Kearnan N , Nowack MK , Schnittger A , Riha K . 2010 Meiotic progression in Arabidopsis is governed by complex regulatory interactions between SMG7, TDM1, and the meiosis I‐specific cyclin TAM. Plant Cell 22: 3791–3803. 21119056 10.1105/tpc.110.078378 PMC3015126

[nph16014-bib-0006] Cai X , Dong FG , Edelmann RE , Makaroff CA . 2003 The Arabidopsis SYN1 cohesin protein is required for sister chromatid arm cohesion and homologous chromosome pairing. Journal of Cell Science 116: 2999–3007. 12783989 10.1242/jcs.00601

[nph16014-bib-0007] Capron A , Serralbo O , Fülöp K , Frugier F , Parmentier Y , Dong A , Lecureuil A , Guerche P , Kondorosi E , Scheres B *et al* 2003 The Arabidopsis anaphase‐promoting complex or cyclosome: molecular and genetic characterization of the APC2 subunit. Plant Cell 15: 2370–2382. 14508008 10.1105/tpc.013847 PMC197302

[nph16014-bib-0008] Chan GK , Liu ST , Yen TJ . 2005 Kinetochore structure and function. Trends in Cell Biology 15: 589–598. 16214339 10.1016/j.tcb.2005.09.010

[nph16014-bib-0009] Chelysheva L , Grandont L , Vrielynck N , le Guin S , Mercier R , Grelon M . 2010 An easy protocol for studying chromatin and recombination protein dynamics during *Arabidopsis thaliana* meiosis: immunodetection of cohesins, histones and MLH1. Cytogenetic and Genome Research 129: 143–153. 20628250 10.1159/000314096

[nph16014-bib-0010] Cifuentes M , Jolivet S , Cromer L , Harashima H , Bulankova P , Renne C , Crismani W , Nomura Y , Nakagami H , Sugimoto K *et al* 2016 TDM1 regulation determines the number of meiotic divisions. PLoS Genetics 12: e1005856. 26871453 10.1371/journal.pgen.1005856 PMC4752240

[nph16014-bib-0011] Cooper KF , Mallory MJ , Egeland DB , Jarnik M , Strich R . 2000 Ama1p is a meiosis‐specific regulator of the anaphase promoting complex/cyclosome in yeast. Proceedings of the National Academy of Sciences, USA 97: 14548–14553. 10.1073/pnas.250351297 PMC18956 11114178

[nph16014-bib-0012] Cromer L , Heyman J , Touati S , Harashima H , Araou E , Girard C , Horlow C , Wassmann K , Schnittger A , De Veylder L *et al* 2012 OSD1 promotes meiotic progression via APC/C inhibition and forms a regulatory network with TDM and CYCA1;2/TAM. PLoS Genetics 8: e1002865. 22844260 10.1371/journal.pgen.1002865 PMC3406007

[nph16014-bib-0013] Cromer L , Jolivet S , Horlow C , Chelysheva L , Heyman J , De Jaeger G , Koncz C , De Veylder L , Mercier R . 2013 Centromeric cohesion is protected twice at meiosis, by SHUGOSHINs at anaphase 1 and by PATRONUS at interkinesis. Current Biology 23: 2090–2099. 24206843 10.1016/j.cub.2013.08.036

[nph16014-bib-0014] Cullen CF , May KM , Hagan IM , Glover DM , Ohkura H . 2000 A new genetic method for isolating functionally interacting genes: high plo1^+^‐dependent mutants and their suppressors define genes in mitotic and septation pathways in fission yeast. Genetics 155: 1521–1534. 10924454 10.1093/genetics/155.4.1521 PMC1461180

[nph16014-bib-0015] Di Fiore B , Wurzenberger C , Davey NE , Pines J . 2016 The mitotic checkpoint complex requires an evolutionary conserved cassette to bind and inhibit active APC/C. Molecular Cell 64: 1144–1153. 27939943 10.1016/j.molcel.2016.11.006 PMC5179498

[nph16014-bib-0016] Duro E , Marston AL . 2015 From equator to pole: splitting chromosomes in mitosis and meiosis. Genes & Development 29: 109–122. 25593304 10.1101/gad.255554.114 PMC4298131

[nph16014-bib-0017] Eloy NB , Lima MD , Van Damme D , Vanhaeren H , Gonzalez N , De Milde L , Hemerly AS , Beemster GTS , Inze D , Ferreira PCG . 2011 The APC/C subunit 10 plays an essential role in cell proliferation during leaf development. The Plant Journal 68: 351–363. 21711400 10.1111/j.1365-313X.2011.04691.x

[nph16014-bib-0018] d'Erfurth I , Cromer L , Jolivet S , Girard C , Horlow C , Sun YJ , To JPC , Berchowitz LE , Copenhaver GP , Mercier R . 2010 The CYCLIN‐A CYCA1;2/TAM is required for the meiosis I to meiosis II transition and cooperates with OSD1 for the prophase to first meiotic division transition. PLoS Genetics 6: e1000989. 20585549 10.1371/journal.pgen.1000989 PMC2887465

[nph16014-bib-0019] Fülöp K , Tarayre S , Kelemen Z , Horvath G , Kevei Z , Nikovics K , Bako L , Brown S , Kondorosi A , Kondorosi E . 2005 Arabidopsis anaphase‐promoting complexes: multiple activators and wide range of substrates might keep APC perpetually busy. Cell Cycle 4: 1084–1092. 15970679

[nph16014-bib-0020] Gao J , Yang S , Cheng W , Fu Y , Leng J , Yuan X , Jiang N , Ma J , Feng X . 2017 GmILPA1, encoding an APC8‐like protein, controls leaf petiole angle in soybean. Plant Physiology 174: 1167–1176. 28336772 10.1104/pp.16.00074 PMC5462013

[nph16014-bib-0021] Garbe D , Doto JB , Sundaram MV . 2004 *Caenorhabditis elegans* lin‐35/Rb, efl‐1/E2F and other synthetic multivulva genes negatively regulate the anaphase‐promoting complex gene mat‐3/APC8. Genetics 167: 663–672. 15238519 10.1534/genetics.103.026021 PMC1470888

[nph16014-bib-0022] Guo L , Jiang L , Zhang Y , Lu XL , Xie Q , Weijers D , Liu CM . 2016 The anaphase‐promoting complex initiates zygote division in Arabidopsis through degradation of cyclin B1. The Plant Journal 86: 161–174. 26952278 10.1111/tpj.13158

[nph16014-bib-0023] Hellmuth S , Pohlmann C , Brown A , Bottger F , Sprinzl M , Stemmann O . 2015 Positive and negative regulation of vertebrate separase by Cdk1‐cyclin B1 may explain why securin is dispensable. Journal of Biological Chemistry 290: 8002–8010. 25659430 10.1074/jbc.M114.615310 PMC4367298

[nph16014-bib-0024] Heyman J , De Veylder L . 2012 The anaphase‐promoting complex/cyclosome in control of plant development. Molecular Plant 5: 1182–1194. 23034505 10.1093/mp/sss094

[nph16014-bib-0025] Holt JE , Pye V , Boon E , Stewart JL , Garcia‐Higuera I , Moreno S , Rodriguez R , Jones KT , McLaughlin EA . 2014 The APC/C activator FZR1 is essential for meiotic prophase I in mice. Development 141: 1354–1365. 24553289 10.1242/dev.104828

[nph16014-bib-0026] Homer H . 2013 The APC/C in female mammalian meiosis I. Reproduction 146: R61–R71. 23687281 10.1530/REP-13-0163

[nph16014-bib-0027] Ji W , Luo Y , Ahmad E , Liu S . 2018 Direct interactions of mitotic arrest deficient 1 (MAD1) domains with each other and MAD2 conformers are required for mitotic checkpoint signaling. Journal of Biological Chemistry 293: 484–496. 29162720 10.1074/jbc.RA117.000555 PMC5767855

[nph16014-bib-0028] Jin F , Hamada M , Malureanu L , Jeganathan KB , Zhou W , Morbeck DE , van Deursen JM . 2010 Cdc20 is critical for meiosis I and fertility of female mice. PLoS Genetics 6: e1001147. 20941357 10.1371/journal.pgen.1001147 PMC2947999

[nph16014-bib-0029] Kashevsky H , Wallace JA , Reed BH , Lai C , Hayashi‐Hagihara A , Orr‐Weaver TL . 2002 The anaphase promoting complex/cyclosome is required during development for modified cell cycles. Proceedings of the National Academy of Sciences, USA 99: 11217–11222. 10.1073/pnas.172391099 PMC123236 12169670

[nph16014-bib-0030] Kelley LA , Mezulis S , Yates CM , Wass MN , Sternberg MJ . 2015 The Phyre2 web portal for protein modeling, prediction and analysis. Nature Protocols 10: 845–858. 25950237 10.1038/nprot.2015.053 PMC5298202

[nph16014-bib-0031] Kevei Z , Baloban M , Da Ines O , Tiricz H , Kroll A , Regulski K , Mergaert P , Kondorosi E . 2011 Conserved CDC20 cell cycle functions are carried out by two of the five isoforms in *Arabidopsis thaliana* . PLoS ONE 6: e20618. 21687678 10.1371/journal.pone.0020618 PMC3110789

[nph16014-bib-0032] Kimata Y , Kitamura K , Fenner N , Yamano H . 2011 Mes1 controls the meiosis I to meiosis II transition by distinctly regulating the anaphase‐promoting complex/cyclosome coactivators Fzr1/Mfr1 and Slp1 in fission yeast. Molecular Biology of the Cell 22: 1486–1494. 21389117 10.1091/mbc.E10-09-0774 PMC3084671

[nph16014-bib-0033] Klein F , Mahr P , Galova M , Buonomo SBC , Michaelis C , Nairz K , Nasmyth K . 1999 A central role for cohesins in sister chromatid cohesion, formation of axial elements, and recombination during yeast meiosis. Cell 98: 91–103. 10412984 10.1016/S0092-8674(00)80609-1

[nph16014-bib-0034] Komaki S , Schnittger A . 2017 The spindle assembly checkpoint in Arabidopsis is rapidly shut off during severe stress. Developmental Cell 43: 172–185. 29065308 10.1016/j.devcel.2017.09.017

[nph16014-bib-0035] Kwee HS , Sundaresan V . 2003 The NOMEGA gene required for female gametophyte development encodes the putative APC6/CDC16 component of the Anaphase Promoting Complex in Arabidopsis. The Plant Journal 36: 853–866. 14675450 10.1046/j.1365-313x.2003.01925.x

[nph16014-bib-0036] Lam WS , Yang XH , Makaroff CA . 2005 Characterization of *Arabidopsis thaliana* SMC1 and SMC3: evidence that AtSMC3 may function beyond chromosome cohesion. Journal of Cell Science 118: 3037–3048. 15972315 10.1242/jcs.02443

[nph16014-bib-0037] McGuinness BE , Anger M , Kouznetsova A , Gil‐Bernabe AM , Helmhart W , Kudo NR , Wuensche A , Taylor S , Hoog C , Novak B *et al* 2009 Regulation of APC/C activity in oocytes by a Bub1‐dependent spindle assembly checkpoint. Current Biology 19: 369–380. 19249208 10.1016/j.cub.2009.01.064

[nph16014-bib-0038] Nagasaka K , Gallego‐Paez LM , Hirota T . 2011 Mitosis and meiosis: molecular control of chromosome separation. In: eLS. Chichester, UK: John Wiley & Sons, Ltd.

[nph16014-bib-0039] Niu BX , Wang LD , Zhang LS , Ren D , Ren R , Copenhaver GP , Ma H , Wang YX . 2015 Arabidopsis cell division cycle 20.1 is required for normal meiotic spindle assembly and chromosome segregation. Plant Cell 27: 3367–3382. 26672070 10.1105/tpc.15.00834 PMC4707457

[nph16014-bib-0040] Okaz E , Arguello‐Miranda O , Bogdanova A , Vinod PK , Lipp JJ , Markova Z , Zagoriy I , Novak B , Zachariae W . 2012 Meiotic prophase requires proteolysis of M phase regulators mediated by the meiosis‐specific APC/CAma1. Cell 151: 603–618. 23101628 10.1016/j.cell.2012.08.044

[nph16014-bib-0041] Pal M , Nagy O , Menesi D , Udvardy A , Deak P . 2007 Structurally related TPR subunits contribute differently to the function of the anaphase‐promoting complex in *Drosophila melanogaster* . Journal of Cell Science 120: 3238–3248. 17878237 10.1242/jcs.004762

[nph16014-bib-0042] Perez‐Perez JM , Serralbo O , Vanstraelen M , Gonzalez C , Criqui MC , Genschik P , Kondorosi E , Scheres B . 2008 Specialization of CDC27 function in the *Arabidopsis thaliana* anaphase‐promoting complex (APC/C). The Plant Journal 53: 78–89. 17944809 10.1111/j.1365-313X.2007.03312.x

[nph16014-bib-0043] Pesin JA , Orr‐Weaver TL . 2008 Regulation of APC/C activators in mitosis and meiosis. Annual Review of Cell and Developmental Biology 24: 475–499. 10.1146/annurev.cellbio.041408.115949 PMC4070676 18598214

[nph16014-bib-0044] Peter M , Castro A , Lorca T , Le Peuch C , Magnaghi‐Jaulin L , Doree M , Labbe JC . 2001 The APC is dispensable for first meiotic anaphase in *Xenopus* oocytes. Nature Cell Biology 3: 83–87. 11146630 10.1038/35050607

[nph16014-bib-0045] Peters JM . 2002 The anaphase‐promoting complex: proteolysis in mitosis and beyond. Molecular Cell 9: 931–943. 12049731 10.1016/s1097-2765(02)00540-3

[nph16014-bib-0046] Peters JM . 2006 The anaphase promoting complex/cyclosome: a machine designed to destroy. Nature Reviews Molecular Cell Biology 7: 644–656. 16896351 10.1038/nrm1988

[nph16014-bib-0047] Peterson R , Slovin JP , Chen C . 2010 A simplified method for differential staining of aborted and nonaborted pollen grains. International Journal of Plant Biology 1: e13.

[nph16014-bib-0048] Pines J . 2011 Cubism and the cell cycle: the many faces of the APC/C. Nature Reviews Molecular Cell Biology 12: 427–438. 21633387 10.1038/nrm3132

[nph16014-bib-0049] Qiao R , Weissmann F , Yamaguchi M , Brown NG , VanderLinden R , Imre R , Jarvis MA , Brunner MR , Davidson IF , Litos G *et al* 2016 Mechanism of APC/CCDC20 activation by mitotic phosphorylation. Proceedings of the National Academy of Sciences, USA 113: E2570–E2578. 10.1073/pnas.1604929113 PMC4868491 27114510

[nph16014-bib-0050] Rankin S , Dawson DS . 2016 Recent advances in cohesin biology. F1000Research 5. doi: 10.12688/f1000research.8881.1. 10.12688/f1000research.8881.1 PMC4975370 27547382

[nph16014-bib-0051] Sarangapani KK , Duro E , Deng Y , Alves FD , Ye QZ , Opoku KN , Ceto S , Rappsilber J , Corbett KD , Biggins S *et al* 2014 Sister kinetochores are mechanically fused during meiosis I in yeast. Science 346: 248–251. 25213378 10.1126/science.1256729 PMC4226495

[nph16014-bib-0052] Schmittgen TD , Livak KJ . 2008 Analyzing real‐time PCR data by the comparative C(T) method. Nature Protocols 3: 1101–1108. 18546601 10.1038/nprot.2008.73

[nph16014-bib-0053] Schreiber A , Stengel F , Zhang Z , Enchev RI , Kong EH , Morris EP , Robinson CV , da Fonseca PC , Barford D . 2011 Structural basis for the subunit assembly of the anaphase‐promoting complex. Nature 470: 227–232. 21307936 10.1038/nature09756

[nph16014-bib-0054] Severson AF , von Dassow G , Bowerman B . 2016 Oocyte meiotic spindle assembly and function. Current Topics in Developmental Biology 116: 65–98. 26970614 10.1016/bs.ctdb.2015.11.031 PMC7744206

[nph16014-bib-0055] Singh DK , Spillane C , Siddiqi I . 2015 PATRONUS1 is expressed in meiotic prophase I to regulate centromeric cohesion in Arabidopsis and shows synthetic lethality with OSD1. BMC Plant Biology 15: 201. 26272661 10.1186/s12870-015-0558-6 PMC4536785

[nph16014-bib-0056] Sivakumar S , Gorbsky GJ . 2015 Spatiotemporal regulation of the anaphase‐promoting complex in mitosis. Nature Reviews Molecular Cell Biology 16: 82–94. 25604195 10.1038/nrm3934 PMC4386896

[nph16014-bib-0057] Wang Y , Cheng Z , Huang J , Shi Q , Hong Y , Copenhaver GP , Gong ZZ , Ma H . 2012a The DNA replication factor RFC1 is required for interference‐sensitive meiotic crossovers in *Arabidopsis thaliana* . PLoS Genetics 8: e1003039. 23144629 10.1371/journal.pgen.1003039 PMC3493451

[nph16014-bib-0058] Wang Y , Cheng Z , Lu P , Timofejeva L , Ma H . 2014 Molecular cell biology of male meiotic chromosomes and isolation of male meiocytes in *Arabidopsis thaliana* . Methods in Molecular Biology 1110: 217–230. 24395259 10.1007/978-1-4614-9408-9_10

[nph16014-bib-0059] Wang Y , Hou YN , Gu HY , Kang DM , Chen ZL , Liu JJ , Qu LJ . 2012b The Arabidopsis APC4 subunit of the anaphase‐promoting complex/cyclosome (APC/C) is critical for both female gametogenesis and embryogenesis. The Plant Journal 69: 227–240. 21910774 10.1111/j.1365-313X.2011.04785.x

[nph16014-bib-0060] Wang YB , Hou YN , Gu HY , Kang DM , Chen ZL , Liu JJ , Qu LJ . 2013 The Arabidopsis anaphase‐promoting complex/cyclosome subunit 1 is critical for both female gametogenesis and embryogenesis. Journal of Integrative Plant Biology 55: 64–74. 23206231 10.1111/jipb.12018

[nph16014-bib-0061] Wang J , Niu B , Huang J , Wang H , Yang X , Dong A , Makaroff C , Ma H , Wang Y . 2016 The PHD finger protein MMD1/DUET ensures the progression of male meiotic chromosome condensation and directly regulates the expression of the condensin gene CAP‐D3. Plant Cell 28: 1894–1909. 27385818 10.1105/tpc.16.00040 PMC5006699

[nph16014-bib-0062] Watanabe Y . 2004 Modifying sister chromatid cohesion for meiosis. Journal of Cell Science 117: 4017–4023. 15316077 10.1242/jcs.01352

[nph16014-bib-0063] Waterhouse A , Bertoni M , Bienert S , Studer G , Tauriello G , Gumienny R , Heer FT , de Beer TAP , Rempfer C , Bordoli L *et al* 2018 SWISS‐MODEL: homology modelling of protein structures and complexes. Nucleic Acids Research 46: W296–W303. 29788355 10.1093/nar/gky427 PMC6030848

[nph16014-bib-0064] Yamashita YM , Nakaseko Y , Kumada K , Nakagawa T , Yanagida M . 1999 Fission yeast APC/cyclosome subunits, Cut20/Apc4 and Cut23/Apc8, in regulating metaphase‐anaphase progression and cellular stress responses. Genes to Cells 4: 445–463. 10526233 10.1046/j.1365-2443.1999.00274.x

[nph16014-bib-0065] Yu HT , Peters JM , King RW , Page AM , Hieter P , Kirschner MW . 1998 Identification of a cullin homology region in a subunit of the anaphase‐promoting complex. Science 279: 1219–1222. 9469815 10.1126/science.279.5354.1219

[nph16014-bib-0066] Zamariola L , De Storme N , Vannerum K , Vandepoele K , Armstrong SJ , Franklin FCH , Geelen D . 2014a SHUGOSHINs and PATRONUS protect meiotic centromere cohesion in *Arabidopsis thaliana* . The Plant Journal 77: 782–794. 24506176 10.1111/tpj.12432

[nph16014-bib-0067] Zamariola L , Tiang CL , De Storme N , Pawlowski W , Geelen D . 2014b Chromosome segregation in plant meiosis. Frontiers in Plant Science 5. doi: 10.3389/fpls.2014.00279. 10.3389/fpls.2014.00279 PMC4060054 24987397

[nph16014-bib-0068] Zhang JF , Wan LX , Dai XP , Sun Y , Wei WY . 2014 Functional characterization of Anaphase Promoting Complex/Cyclosome (APC/C) E3 ubiquitin ligases in tumorigenesis. Biochimica Et Biophysica Acta–Reviews on Cancer 1845: 277–293. 10.1016/j.bbcan.2014.02.001 PMC3995847 24569229

[nph16014-bib-0069] Zheng BL , Chen XM , McCormick S . 2011 The anaphase‐promoting complex is a dual integrator that regulates both MicroRNA‐mediated transcriptional regulation of Cyclin B1 and degradation of Cyclin B1 during Arabidopsis male gametophyte development. Plant Cell 23: 1033–1046. 21441434 10.1105/tpc.111.083980 PMC3082252

