# Supplementary material for: The Arabidopsis anaphase‐promoting complex/cyclosome subunit 8 is required for male meiosis
Source: New Phytol. 2019 Jul 24;224(1):229–41. doi: 10.1111/nph.16014 (PMC6771777; doi:10.1111/nph.16014)
Supplement: Supplementary file 1 — Fig. S1 Silique images and seed numbers in wild‐type, atapc mutants and the APC8‐complementated plants. Fig. S2 Relative transcript levels of AtAPC8, AtOSD1, AtTDM1 and AtPANS1 in wild‐type (Col‐0), atapc8‐1 and APC8‐complementated plant. Fig. S3 FISH analysis of meiotic chromosomes in APC8‐complementated plants using a centromere probe. Fig. S4 Mitotic chromosome behaviours in wild‐type, atapc8‐1 and APC8‐complementated plants. Fig. S5 Immunolocalisation of AtSMC3 in wild‐type and atapc8‐1. Fig. S6 Immunolocalisation of microtubules during mitosis in wild‐type and atapc8‐1 mutant. Fig. S7 Examination of protein−protein interaction by yeast two‐hybrid assay and modelling the APC8 protein structure. Table S1 Primers used in this study. [file NPH-224-229-s001.pdf]

***New Phytologist* Supporting Information Figures S1-S7 and Table S1**

Article title: **The Arabidopsis anaphase promoting complex/cyclosome subunit 8 is required for male meiosis**

Authors: **Rong-Yan Xu, Jing Xu, Liudan Wang, Baixiao Niu, Gregory P. Copenhaver, Hong Ma, Binglian Zheng, Yingxiang Wang**

Fig. S1. Silique images and seed numbers in WT, *atapc* mutants and the rescued plants.

Fig. S2. Relative transcript levels of *AtAPC8*, *AtOSD1*, *AtTDM1* and *AtPANS1* in wild type (Col-0), *atapc8-1* mutant, and APC8-YFP recused plant.

Fig. S3. FISH analysis of meiotic chromosomes in *apc8-1*–rescued plants using a centromere probe.

Fig. S4. Mitotic chromosome behaviors in WT, *atapc8-1* and *apc8-1* rescued plants.

Fig. S5. Immuno-localization of AtSMC3 in WT and *atapc8-1*.

Fig. S6. Immuno-localization of microtubules during mitosis in WT and *atapc8-1*.

Fig. S7. Examination of protein-protein interaction by Y2H and modeling the APC8 protein structure.

Table S1. Primers used in this study.

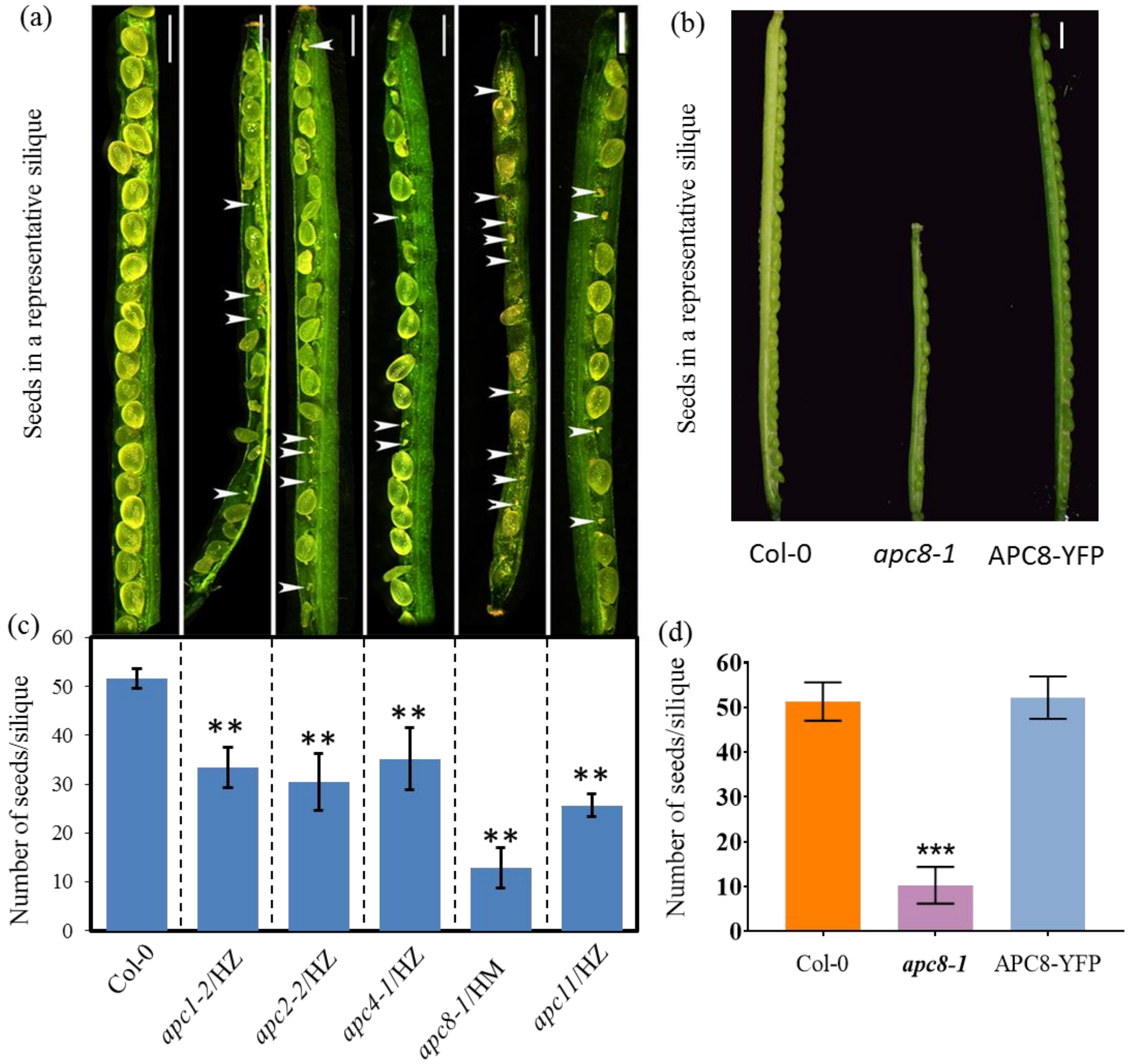

Fig. S1. Silique images and seed numbers in wild type, *atapc* mutants and the rescued plants. (a-b) Arrowheads show shriveled placenta without ovule development; HZ, heterozygote; HM, homozygote, and APC8-YFP rescued plants; Bar=1 mm. (c-d) Seed number per silique in wild type (Col-0), *apc1-2* heterozygote, *apc2-2*/HZ, *apc4-1*/HZ, *apc8-1*/HM and *apc11*/HZ plants. HZ, heterozygote; HM, homozygote. Error bars represent SD for data as described. *P ≤0.05,**P ≤0.01, ***P ≤ 0.001, Two-tailed Student's t test.

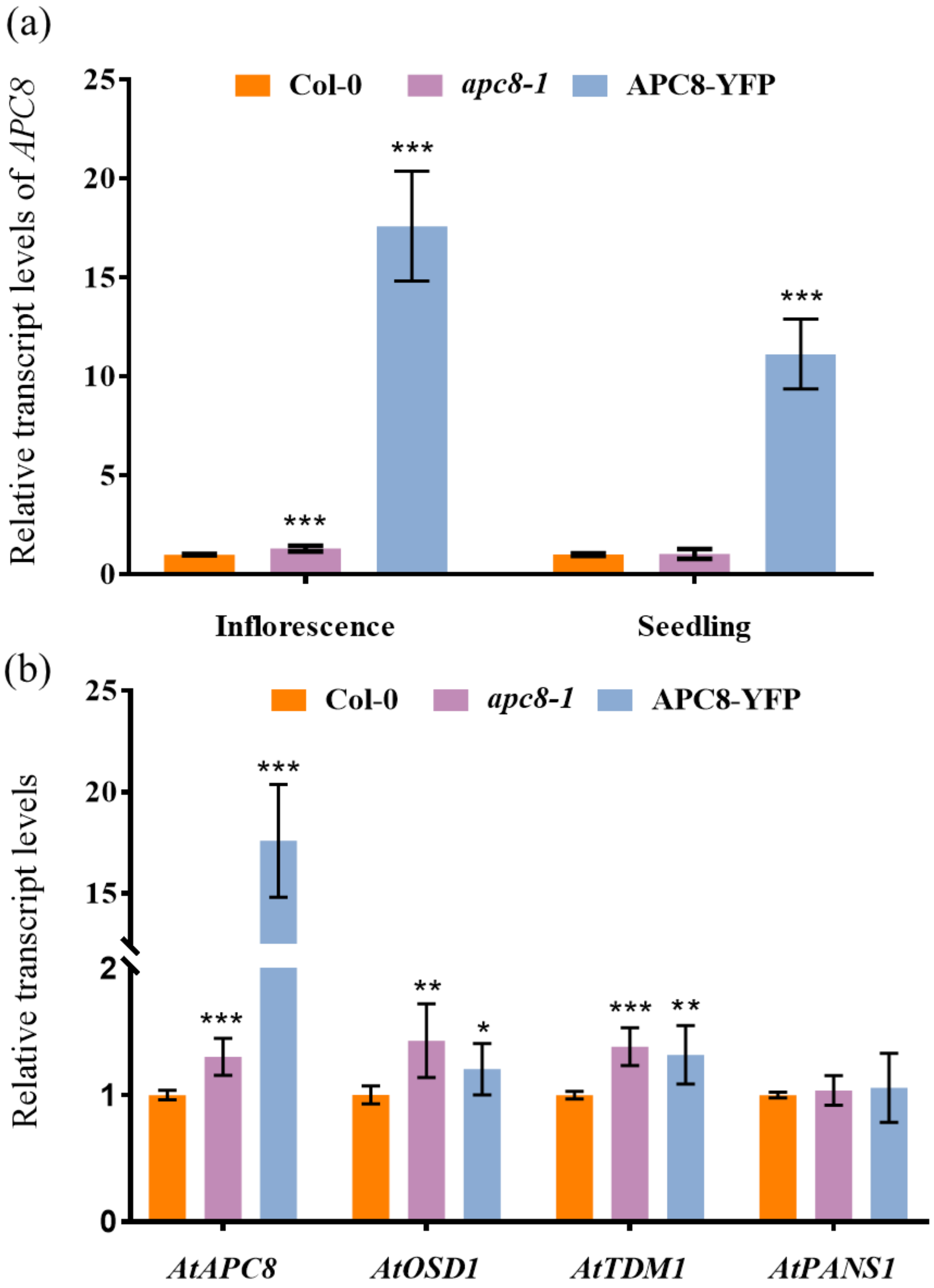

Fig. S2. Relative transcript levels of *AtAPC8*, *AtOSD1*, *AtTDM1* and *AtPANS1* in wild type (Col-0), *atapc8-1* mutant, and APC8-YFP recused plant.

(a) The expression levels of *APC8* gene in inflorescences and seedlings of wild type (Col-0), *apc8-1* mutant, APC8-YFP recused plant. (b) Relative transcript levels of *APC8*, *OSD1*, *TDM1* and *PANS1* in inflorescences of wild type (Col-0), *apc8-1* mutant, APC8-YFP recused plant. Error bars represent SD for data as described. *P ≤0.05,**P ≤0.01, ***P ≤ 0.001, Two-tailed Student's t test.

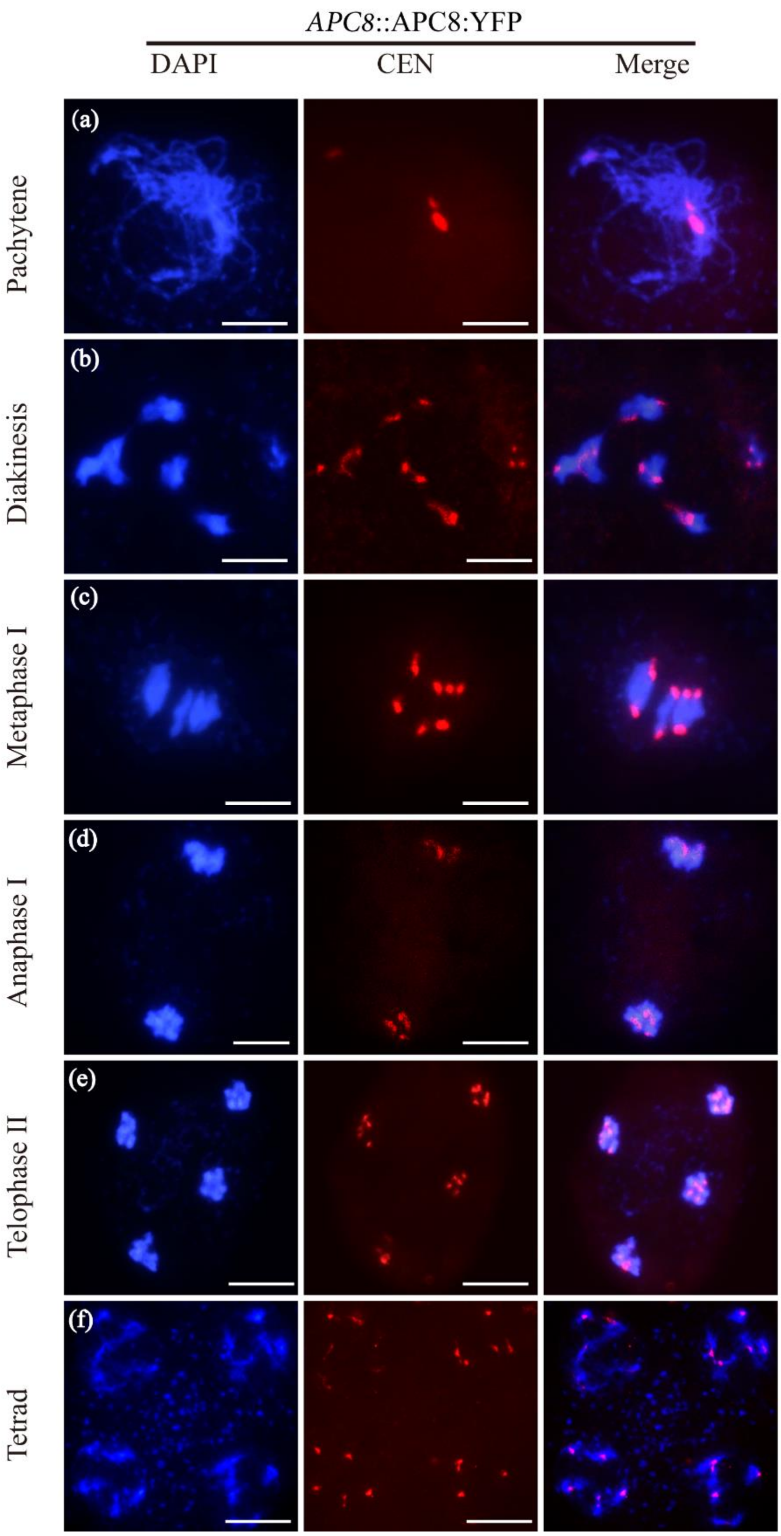

Fig. S3. Chromosomal phenotype of APC8::APC8:YFP meiocytes using a centromere probe.

Chromosomes were spread and hybridized with a centromere DNA probe. (a) Pachytene; (b) Diakinesis; (c) Metaphase Ⅰ; (d) Anaphase Ⅰ; (e) Telophase Ⅱ; (f) Tetrad stages. Blue images show chromosomes stained with DAPI; Red spots show DNA signals at centromeres. All pictures were taken under a 100 × objective of a fluorescence microscope (Zeiss Axio Imager), Bar = 5 μm.

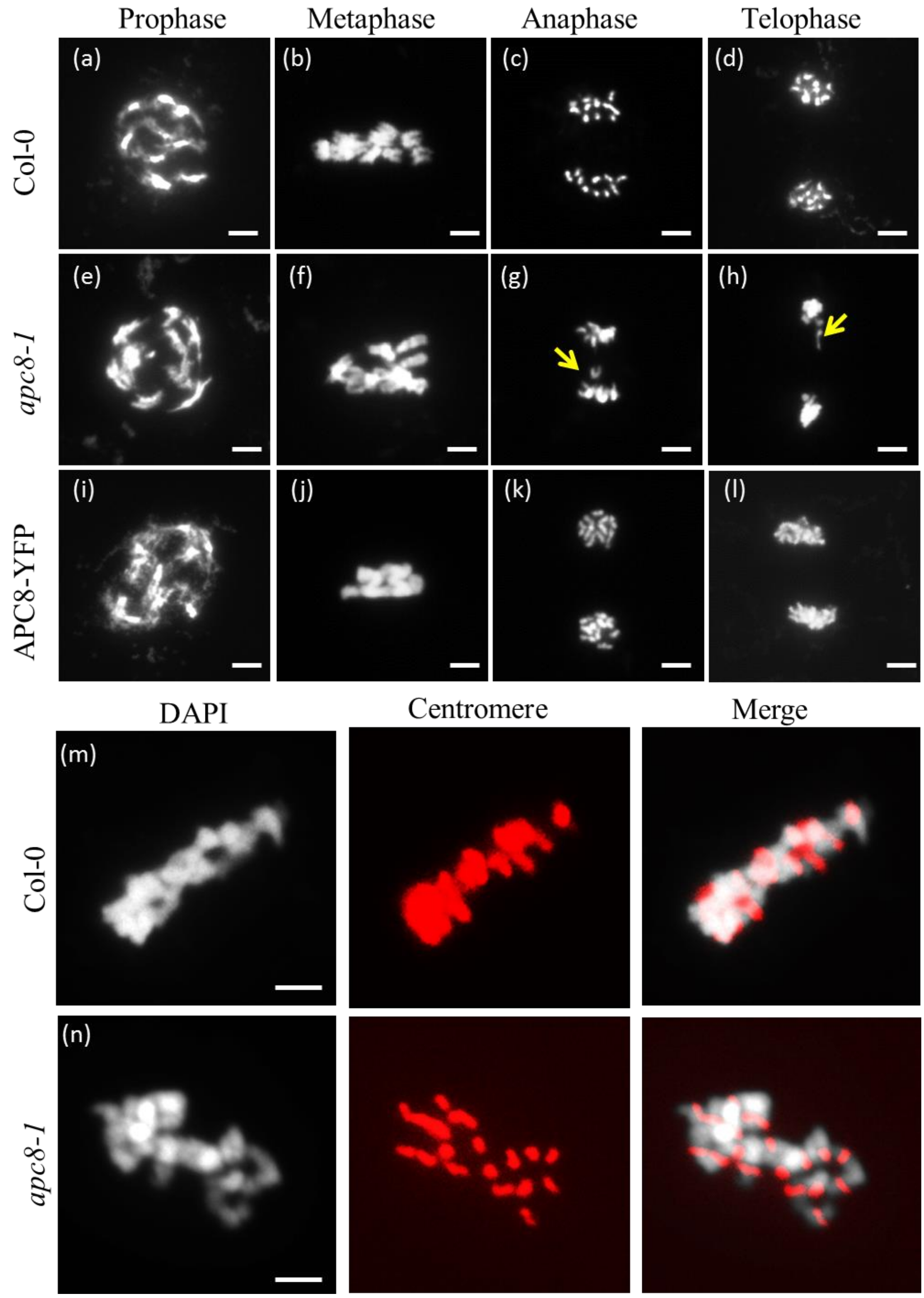

Fig. S4. Mitotic chromosome behaviors in root tips of WT, *atapc8-1* mutant, and APC8-YFP rescued plants.

Mitotic chromosome spread was prepared from root tips of examined plants. (a-d) WT; (e-h) *apc8-1* mutant, (i-l) APC8-YFP rescued plants; (a, e, i) Prophase; (b, f, j) Metaphase; (c, g, k) Anaphase; and (d, h, l) Telophase. Arrowheads in (g, h) show lagging chromosomes in the *apc8-1* mutant. (m-n) FISH analyses of metaphase I chromosomes in WT and *apc8-1* with centromere probe. All photographs were taken using a 100 × objective of a fluorescence microscope (Zeiss Axio Imager), bar = 5 μm.

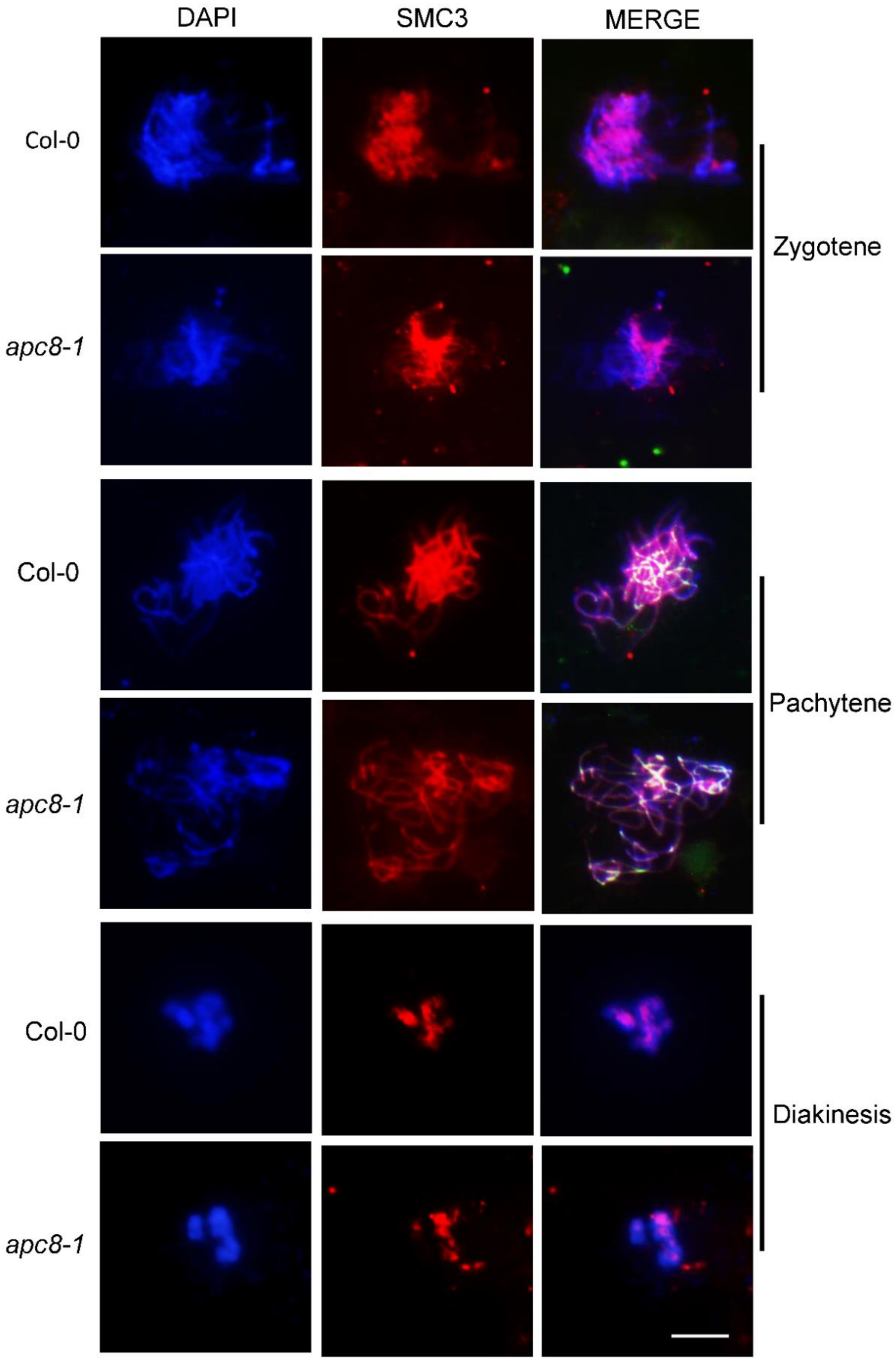

Fig. S5. Immuno-localization of SMC3 in wild type and *atapc8-1*.
Chromosomes are stained in blue by DAPI (left panel), in red by anti-SMC3 antibody (middle panel). The right panel is merged by left and middle panel. All pictures were taken under a 100 × objective of a fluorescence microscope (Zeiss Axio Imager), Bar = 5 μm.

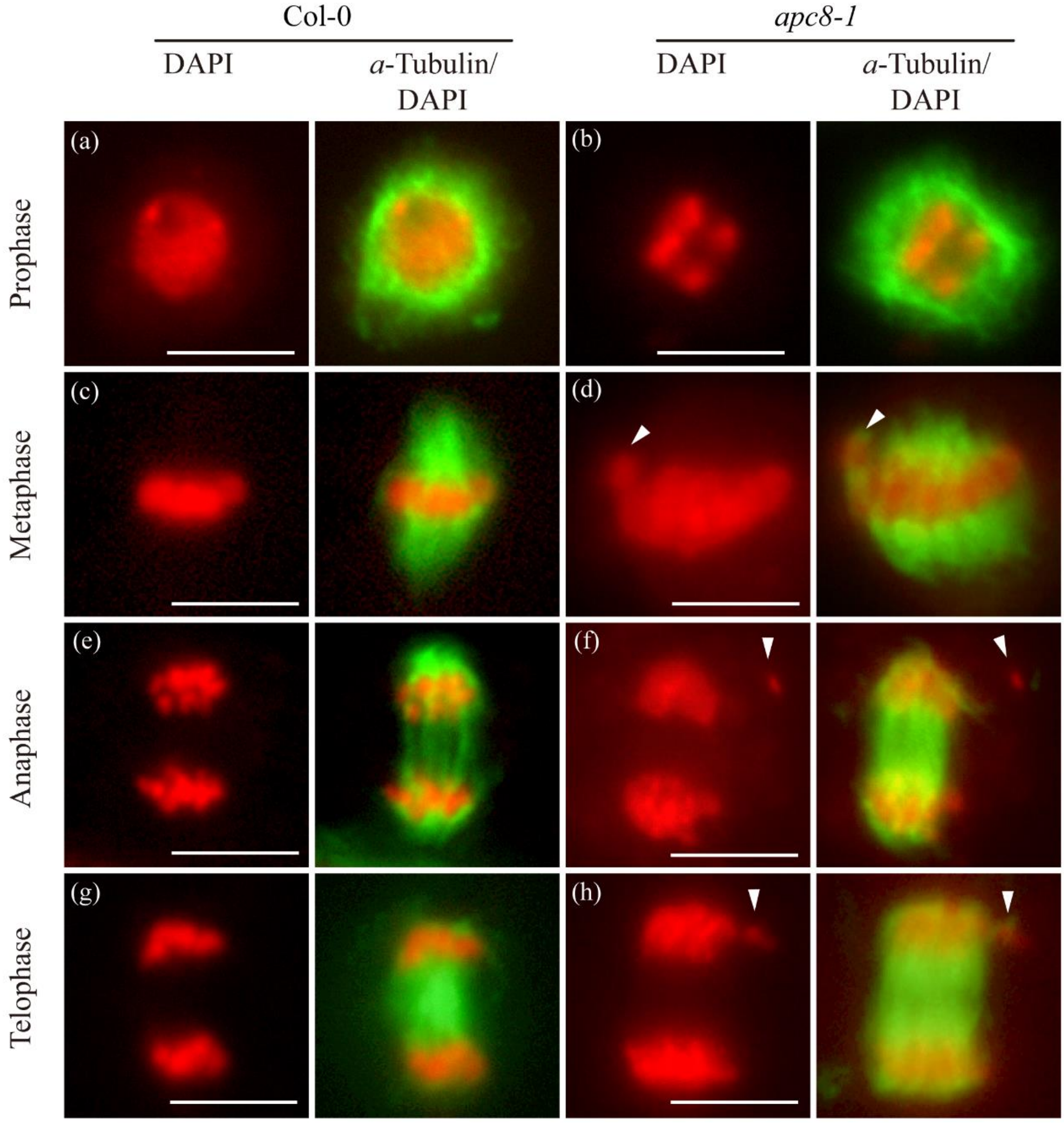

Fig. S6. Immuno-localization of microtubules in mitosis in WT and *atapc8-1*.
In WT mitotic cells, microtubules are observed surrounding the nucleus in prophase and they are organized into a typical spindle at metaphase. With microtubules of the spindle pulling towards the opposite poles, chromosomes separate at anaphase. In contrast, the abnormal microtubule organization is observed in *apc8-1* and subsequent formation of improperly aligned chromosomes. WT (a, c, e, g) and *apc8-1* mutant (b, d, f, h). All photographs were taken using a 100 × objective of a fluorescence microscope (Zeiss Axio Imager), bar = 5 μm.

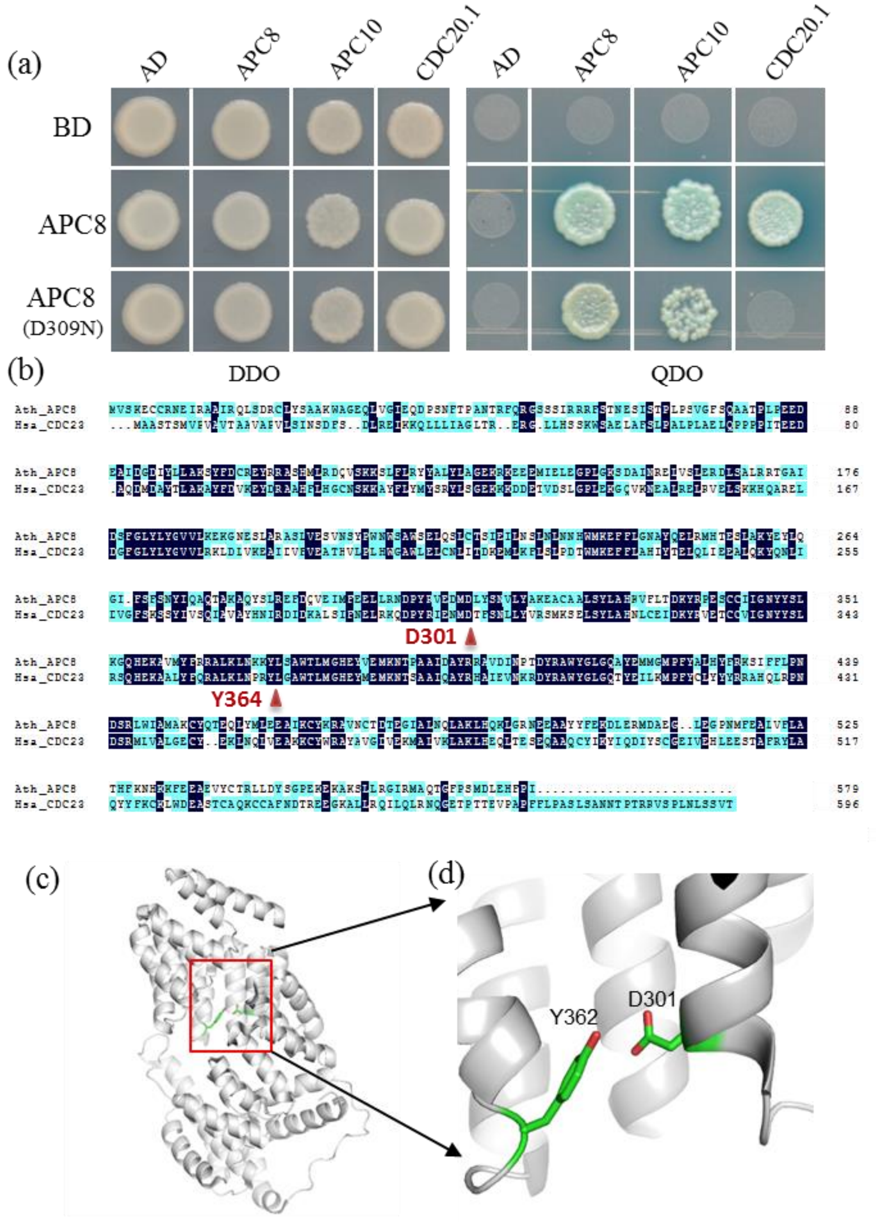

Fig. S7. Examination of protein-protein interaction by Y2H and modeling the APC8 protein structure.

(a) DDO refers SD medium in the absence of Trp and Leu and QDO refers the SD medium without His, Ade, Trp, Leu, and X-α-Gal. (b) Sequence alignment of human CDC23 with Arabidopsis APC8. Red arrowheads show predicted interacting proteins in human. (c) The modeled structure of APC8 (20-500 aa). (d) Regional enlargement from (c). D309 in APC8 is corresponding to D301 in CDC23, while in the 4UI9 structure, D301 and Y364 are closed to each other.

Table S1. Primers used in this study.

Primer name	Primer sequence
**Primers for real-time PCR:**	
ACTIN2-F	CGTACAACCGGTATTGTGCT
ACTIN2-R	TTGATGTCTCTTACAATTTC
APC8-F	TACGTGCGGCTATTCGACAG
APC8-R	CCAACGGAAGGTAACGGTGT
OSD1-F	TGCCCATAGTGACCGCTAG
OSD1-R	GGGATCTCAATAACTCGGCC
TDM1-F	AGGAGGAGACCACCGTATTC
TDM1-R	GCACTATCAACTCGATCTCCAG
PANS1-F	ATGGCGAACATGAACGCTCT
PANS1-R	CGCATGAATCCCAGACTTGT
**Primers for T-DNA mutant genotyping:**	
apc1-2F:	GGGCAATCCAACTTACATGTC
apc1-2R:	CCCATTTGATTCCAAGATTTG
apc2-2F:	GGAGCTTGCCTTGAGAAGTTC
apc2-2R	TCTCCAAGCTTTTCCAGAGTG
apc4-1F:	ACTTGGATCCTTTCAAGAGCC
apc4-1R:	AACTCCATTTTCAGGAATGTAAAG
apc11-F:	TGCGTATCCTTTTGTTGCTTC
apc11-R:	CTTTGTCCAAGCAATCAAACC
**Primers for *apc8-1* point mutant genotyping:**	
APC8-F	CTTTATCTGGCTGGAGAAAA
APC8-R1	CAGATAAATACTTCTTGTTC
**Primers for Y2H**	
APC8-AD-F	GTACCAGATTACGCTCATATGATGGTCTCTAAAGAGTGTTGCCG
APC8-AD-R	GAGCTCGATGGATCCCTAAATAGGAAAATGCTCGAGATCCA
APC8-BD-F	TCAGAGGAGGACCTGCATATGATGGTCTCTAAAGAGTGTTGCCG
APC8-BD-R	CAGGTCGACGGATCCCTAAATAGGAAAATGCTCGAGATCCA
APC8-D309N-F	ATCGGGTAGAAGATATGAATTTGTATTCTAATGTTC
APC8-D309N-R	TTCATATCTTCTACCCGATATGGATCGTT
APC10-F	GGAATTCCATATGATGGCGACAGAGTCATCGG
APC10-R	CGGAATTCTCATCTCAGTGTTGAATAAGTGAG
CDC20.1-F	GGAATTCCATATGATGGATGCAGGTATGAACAACAC
CDC20.1-R	CGGGATCCTTCAACGAATACGATTCACGTGAG
